# Be-SNAP: the Belgian Sepsis National Action Plan

**DOI:** 10.3389/fpubh.2025.1575502

**Published:** 2025-07-01

**Authors:** Annelies Mondelaers, Febe Van de Voorde, Harlinde Peperstraete, Ken Dewitte, Jan De Waele, Ilse Malfait, Patrick Van de Voorde, Erika Vlieghe

**Affiliations:** ^1^Global Health Institute, University of Antwerp, Antwerp, Belgium; ^2^Department of Internal Medicine, Infectious Diseases and Tropical Medicine, Antwerp University Hospital, Antwerp, Belgium; ^3^Department of Emergency Medicine, AZ Maria, Halle, Belgium; ^4^Department of Intensive Care Medicine, Ghent University Hospital, Ghent, Belgium; ^5^Emergency Department, AZ Voorkempen, Malle, Belgium; ^6^Patient Organisation Sepsibel vzw, Oostkamp, Belgium; ^7^Department of Emergency Medicine, Ghent University Hospital, Ghent, Belgium

**Keywords:** sepsis, antimicrobial stewardship, infection prevention and control, public health, early warning, advance care planning, rehabilitation, rapid response

## Abstract

Sepsis represents a significant healthcare challenge in Belgium with an estimated 40,952 cases annually (95% CI 31,938–54,451). This life-threatening condition leads to approximately 7,675 premature deaths per year (95% CI 6,421–9,089) and a loss of 38,106 quality-adjusted life years. The economic impact is substantial with annual costs ranging from €277 million to €4.3 billion. Despite this impact, Belgium lacks a national sepsis plan until present date. Following requests from diverse professional and patient organizations yielding public and political attention, the federal minister of Health requested a scientific advice to be written (November 2023–May 2024), as a basis for a national sepsis plan. This article describes Belgium’s approach to developing a National Sepsis Action Plan, highlighting evidence-based and contextualized key recommendations aimed at reducing the sepsis burden by building on existing initiatives. A multidisciplinary working group was established, including representatives of healthcare workers and professional societies representing relevant disciplines in first, second and tertiary health care settings, home care and long-term care facilities. In addition, input was sought from public health actors and experts (e.g., surveillance, vaccination programs) and patient organizations. A Haddon matrix was made and seven key topics were specified: (1) awareness, (2) prevention, (3) early warning, (4) patient management, (5) post-sepsis rehabilitation, (6) advanced care planning and (7) surveillance and research. For each item, core group members were defined. Each group conducted literature reviews and developed recommendations tailored to the Belgian healthcare system, with consensus achieved during plenary sessions. The final document was externally reviewed by national and international experts. This is the first document addressing comprehensively sepsis prevention and care in Belgium, in its diverse presentations across the community and healthcare system. The next critical steps will involve the establishment of an implementation team and design of a detailed implementation plan.

## 1 Introduction

### 1.1 A national plan to save lives

In 2017, the World Health Assembly (WHA) declared sepsis a priority for global health. It urged member states to develop and implement national strategies for sepsis prevention, diagnosis, and management (1, 2). Seven years after this resolution, 16 countries already prioritized sepsis in their national health policies.

Despite several attempts and the evidence from other countries that coordinated sepsis programs save lives, reduce healthcare costs and improve overall outcomes, Belgium still lacks coordinated action until date. A national action plan should contain an overarching, multidisciplinary, multi-stakeholder approach to prevent or mitigate the burden of sepsis, including the different actors, the resources, tools and structures needed, and the associated political decisions. As such, it aims to provide a roadmap and structured framework for implementation and follow-up of needed interventions, while ensuring synergy with other existing public health programs.

### 1.2 The clinical aspects of sepsis

Sepsis is a life-threatening medical condition that occurs when the body’s response to an infection causes intense inflammation, possibly leading to a profound and dangerous decrease in perfusion (shock) and failure of vital organs (3–5). Sepsis and septic shock are associated with high mortality and substantial, long-lasting morbidity. Depending on the location and population studied, about 25–30% of patients with sepsis die from the condition, while hospital mortality for septic shock approaches 40–60% (6).

Sepsis can develop in anyone, but should be considered more carefully in those at higher risk, such as very young infants, older adult, men, and pregnant women. Persons with chronic conditions, a decreased immune status, a current or recent hospitalization, previous sepsis episodes, limited education levels and living in socio-economically challenging situations also face a higher risk (7–17).

A wide variety of microorganisms can trigger sepsis, including bacteria, fungi, viruses, and parasites. The type of microorganism often depends on the specific region and setting where the infection occurs (18, 19). Of note, in only about 15–25% of sepsis cases the micro-organisms responsible for the disease can be found (through microbiological cultures) and serve as proof of invasive infection (20).

### 1.3 Diagnosing sepsis

Sepsis frequently presents as the clinical deterioration of common and potentially preventable infections. Its early signs may not always be recognized as warning signs by the patient, caretakers and healthcare workers.

Diagnosing sepsis is challenging because no single laboratory or imaging test can unequivocally confirm the condition. Instead, diagnosis relies on a combination of clinical findings and laboratory tests. As a result, sepsis is frequently missed in its early stages. For example, a Dutch study involving 263 patients admitted to an intensive care unit (ICU) revealed that 48.3% of these patients had consulted their general practitioner (GP) in the days prior to their admission. Among these patients, only 64% were referred immediately, and in 43% the GP did not suspect an infection (21).

The current definition is based on the “Sepsis-3 criteria,” introduced by the Third International Consensus Definitions for Sepsis and Septic Shock in 2016. According to these criteria, the key criteria for diagnosing sepsis include the suspicion of infection–based on clinical, laboratory or imaging findings- and evidence of organ failure. Organ failure is defined by a Sequential Organ Failure Assessment (SOFA) score of 2 points or more (4).

Septic shocks is defined as a subset of sepsis, characterized by profound circulatory, cellular, and metabolic abnormalities that significantly increase the risk of mortality. Clinically, patients with septic shock can be identified by a vasopressor requirement to maintain a mean arterial pressure of 65 mmHg or greater along with a serum lactate level exceeding 2 mmol/L (>18 mg/dL) in the absence of hypovolemia.

### 1.4 Treatment of sepsis

Treatment of sepsis and septic shock involves the prompt administration of antibiotics to target the underlying infection, intravenous fluids to maintain blood pressure, and medications to support organ function (22). This often requires intensive medical care, including mechanical ventilation and other life-support measures. Antibiotics are typically started before the specific cause of infection is known (empiric treatment) and subsequently adjusted once test results identify the pathogen (targeted treatment). However, emerging antimicrobial resistance (AMR) poses a challenge, as there may be a mismatch between the empirically chosen antimicrobial and the difficult-to-treat micro-organism, potentially leading to treatment failure and an increased risk of death. Therefore, adequate sepsis management is closely related to the availability of accurate treatment guidelines, effective antimicrobials and strategies to prevent further emergence of AMR (23).

### 1.5 The aftermath of sepsis in sepsis survivors

Sepsis survivors may experience long-term effects, including prolonged organ dysfunction, necrosis of peripheral body parts (e.g., limbs or nose) requiring amputation, neurocognitive impairment, mental health disorders, and an increased risk for new sepsis episodes. In addition, sepsis survivors face an excess hazard of late cardiovascular events, persisting up to 5 years following hospital discharge (24, 25). A cohort study of 116,507 sepsis survivors revealed that 48.9% of survivors experienced one or more adverse outcomes within the first year after sepsis, including new dependency on chronic care, dialysis or respiratory support, and even death (26, 27). As summarized in a World Health Organization (WHO) report, about 1/3 of patients with sepsis die within the first year, 1 out of 6 experience significant morbidity, and 4 of 10 are readmitted within 90 days (28).

### 1.6 The burden of sepsis worldwide

The burden of sepsis is a combination of incidence and clinical outcome (29). However, detailed epidemiological data on sepsis are limited, as it is primarily a clinical diagnosis and not systematically registered in a standardized manner in most countries. The Global Burden of Diseases Study estimated that approximately 49 million cases occur every year worldwide, causing 11 million deaths, with the majority occurring in low- and middle-income settings due to poverty, political corruption, health inequity, and under-resourced and low-resilience public health and acute health care delivery systems. Globally, 20 percent of all deaths are likely caused by sepsis (30). Alarmingly, nearly 3 million children annually die from sepsis, with fatality rates ranging from 19% in developed countries up to 32% in developing countries. The incidence of sepsis appears to be increasing, mainly due to higher survival rates of very low birth weight infants and children with chronic conditions as well as a higher incidence of infections caused by multi-resistant bacteria. Most deaths from sepsis occur within the first days of admission (31), but mortality can also happen months or years later.

The incidence of sepsis in Europe is substantial and increasing due to an aging population. More than 3.4 million individuals develop sepsis in Europe each year, with 700,000 deaths and one-third of survivors dying within a year (19). Variety across European countries is due to differences in healthcare infrastructure, population demographics, and reporting practices.

### 1.7 Sepsis in Belgium

Epidemiologic data on sepsis in Belgium are limited and systematic surveillance data are absent. A recent prospective cohort study described 1,690 episodes of suspected sepsis at the emergency department of a tertiary hospital over a period of 13 months (32). Extrapolating these findings, the annual sepsis cases in Belgium would exceed the number described (*n* = 40,952) in the Global Burden of Diseases Study (30, 33). The only official surveillance data available for Belgium are on healthcare-associated bloodstream infections (HABSI). Between 2013 and 2022, a gradually increasing incidence of HABSI, from 7.8/10,000 hospitalization days in 2013 to 9.2/10,000 in 2022 is described (34).

Sepsis imposes a significant burden on healthcare systems and society. It generates both direct (whether healthcare-related or due to, for instance, adapted education, transportation, housing) and indirect costs (e.g., productivity loss, caregiver impact…) for the patients, family, healthcare providers and insurers, as well as society. Given the lack of proper data, estimating the total cost for an individual sepsis patient and for society is very difficult in Belgium. However, extrapolating from existing national and international literature, it is reasonable to estimate an annual loss of Quality-adjusted Life-years (QALY) for Belgium around 38,000 and the overall annual economic burden likely above 1 billion Euro (range 0.3–4.3 billion).

Addressing the burden of sepsis in Belgium requires urgent, coordinated, and evidence-based action. The lack of systematic surveillance data, combined with the significant human and economic costs, underscores the need for a national sepsis action plan that prioritizes prevention, early recognition, and rapid response. Drawing on global successes and best practices, this plan must establish standardized surveillance systems, promote the implementation of validated tools across care settings, and ensure equitable access to sepsis care for vulnerable populations. By aligning with international initiatives and leveraging multidisciplinary collaboration, Belgium could not only improve patient outcomes and reduce healthcare costs but also inspire other nations facing similar challenges.

## 2 Methodology

In response to the WHA call in 2017, a bottom-up group of sepsis experts from scientific organizations and patient groups submitted a first proposal for a sepsis national action plan in 2021. After extensive deliberation, a core working group was established to finalize the plan in collaboration with various relevant scientific societies and patient representatives. The multidisciplinary group of sepsis experts and patient representatives was selected based on established collaborations in research and patient care. The selection process prioritized individuals and groups who had previously worked together on initiatives related to sepsis. This group was given an official mandate by the Belgian Minister of Health in December 2023. The core writing group defined the scope of the plan and determined the methodology to be followed.

**Figure 1:**
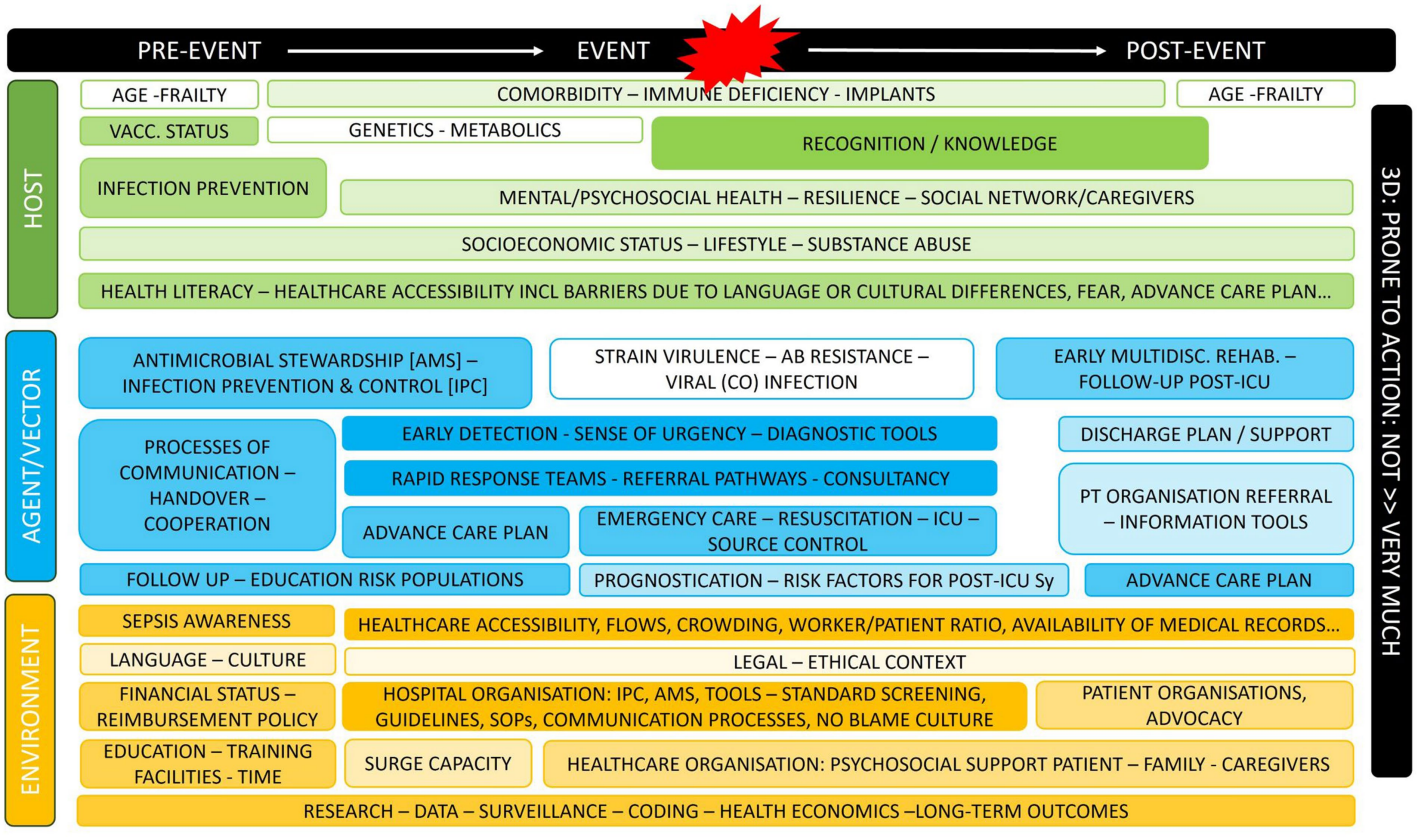
Haddon matrix for sepsis as a public health problem. For each item, core group members also defined how far these were prone to action (more color = more prone).

To further develop the plan, the core group addressed sepsis as a public health issue within the Belgian healthcare system, acknowledging its many parallels with other sudden health emergencies, such as injuries or sudden cardiac arrest, which can lead to severe harm and high costs. Describing such healthcare problems in all its dimensions demands a broad and public health-orientated conceptual framework. The Haddon matrix facilitates this process, aiding in the identification and consideration, beyond mere causality or chronology, of the means available for reducing the undesirable mortality, morbidity, and healthcare costs related to sepsis (35–37). The Haddon Matrix considers three main categories of factors (Host Attributes, Vector or Agent Attributes and Environmental Attributes) across three different phases (pre- to post-event). Each member of the core writing group, in consultation with the groups they represented, wrote out a full Haddon Matrix for the problem of sepsis and these matrixes were then combined into one (Figure 1).

**Figure 2:**
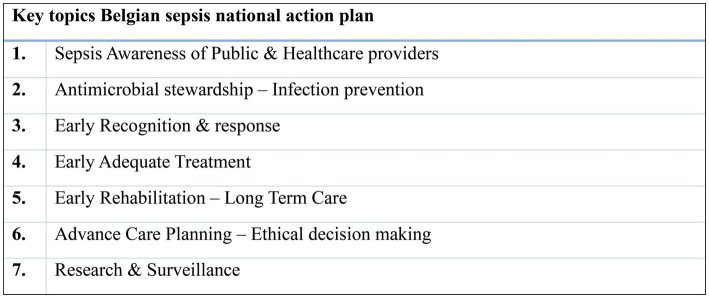
Seven key topics of the Belgian sepsis national action plan.

During a second meeting, seven recurring priority topics within the combined matrix were identified for further exploration, serving as a basis for subsequent recommendations to policy makers and other relevant actors (Figure 2). For each of these seven topics, an extended working group was created with members of the core writing group but also content experts, and/or representatives of other relevant groups and/or societies across the country.

Each working group conducted a thorough literature review to provide evidence of the impact of the topic on important outcome measures and implementation strategies, considering the type of intervention (from a public health perspective) and its respective priority. A Haddon Matrix in itself does not provide prioritization or strategy. Therefore, a more general “public health” approach is necessary. This approach provides a third dimension to the Matrix by evaluating each possible intervention for among others its cost-effectiveness, acceptability, feasibility, and equity (38–40), Literature review was carried out as a rapid review, primarily focusing on systematic reviews, guidelines and/or pivotal clinical studies published after 01/01/2000. Studies included needed to demonstrate a link between the key identified topic and any important outcome. Non-human, non-English, research from low and middle-income countries as well as letters, commentaries and opinions were excluded from this review (41).

Finally, based on their literature evaluation and consensus discussions, the working groups proposed recommendations and provided insights and arguments to support them. These proposals were subsequently discussed during an in-dept one-day conclave. As final step, the draft plan was reviewed by a reading panel of national and international experts in infectious diseases, sepsis, public health and/or healthcare organizations who had not previously been involved in the process.

## 3 Priority topics

### 3.1 Awareness and knowledge on sepsis

Awareness and knowledge of sepsis among both the general public and healthcare professionals (HCPs) are crucial factors in the prevention, early recognition and clinical management of sepsis, yet often lacking. Awareness refers to the mere recognition of sepsis, something which can be raised through large-scale campaigns with easy-to-remember messages (42). Knowledge, on the other hand, also involves understanding and proficiency. It is a more lengthy, structured and nuanced process, that feeds into the professional intuition. Limited evidence exists about the current state of sepsis awareness and early recognition in Belgium. A recent survey conducted by a Belgian sepsis patient representative group, Sepsibel, involved 2,000 participants. The results revealed that two-thirds of respondents had never heard of sepsis before. Only one in five participants understood that sepsis is an extreme reaction of the immune system. However, 70% expressed a desire to learn more about sepsis (43).

#### 3.1.1 Public awareness

Fiest et al. conducted an exhaustive systematic review in 2022 and identified 80 studies that reported on awareness and knowledge of sepsis among patients, the public, or HCPs (nurses, physicians, emergency medical technicians) (44). Overall, public awareness of sepsis appeared quite low, although there were considerable variations among different countries and some indication of gradual improvement over time. In the case of pediatric sepsis, research indicated that up to 33% of sepsis deaths in children could be attributed to delays in seeking medical care by parents (45). Indeed, pediatric sepsis most commonly starts in the community, and the decision and timing of parents in seeking medical care for children contributes significantly to sepsis-related outcomes of children (46, 47).

#### 3.1.2 Healthcare provider awareness

Although HCPs generally have a better awareness and knowledge of sepsis compared to the general public, they may still underestimate its actual mortality risk. HCPs working in hospital settings tend to demonstrate better awareness than their counterparts in prehospital settings (44). Data on sepsis awareness among first-line HCPs are limited. A Dutch observational study found that over one-third of sepsis patients initially assessed by a GP were not referred to a hospital. The highest mortality rates were observed in those in whom the GP did not suspect an infection (21). GPs primarily rely on intuition to diagnose sepsis and are often unfamiliar with more formal criteria for suspected sepsis (48).

Despite their crucial frontline position, there is little evidence regarding sepsis knowledge of other (paramedical) first-line HCPs, such as home care or residential care nurses. Pharmacists could play a key role in informing the public in prehospital settings. In hospitals, the involvement of pharmacists has been associated with reduced time to adequate antibiotic administration (49).

#### 3.1.3 Interventions to raise public awareness

Several international initiatives, such as the Surviving Sepsis Campaign and the World Sepsis Day (Global Sepsis Alliance), have gradually increased overall sepsis awareness over the past three decades (44). Additionally, several countries have already implemented large-scale promotion campaigns to improve sepsis awareness such as Think Sepsis! and Just Ask “Could it be sepsis?.” While the impact of such interventions remains uncertain, evidence from other major health problems, like stroke, suggests a clear potential for improvement in early recognition and overall outcome (44, 50–54).

Promotion campaigns should combine general understandable messages for the public and targeted messages for high-risk populations, e.g., immunosuppressed individuals and children at-risk. There should be special attention to different languages and hard-to-reach groups. While broad campaigns may generate more visibility and overall support, they may also lead to overconsumption and increased anxiety. It is therefore important that campaigns are carefully planned, in synergy with other pre-existing initiatives [such as antimicrobial stewardship (AMS)] and integrated into a broader public health strategy. It is crucial to evaluate their impact as part of an audit cycle considering both short- and long-term effects.

Specifically targeted campaigns should be developed, focusing on sepsis in children. Fever is common in children and the early signs of sepsis can be challenging for parents and caregivers to recognize. Again, while it is vital to avoid late recognition of sepsis, overtreatment should be prevented. To support this, an easy-to-use triage tool for caregivers and healthcare providers -similar to the “red flag” system from the National Institute for Health and Care Excellence (NICE UK) (55) or the Perth Children’s Hospital Escalation System- should be implemented and actively promoted.

Key public information sources include mass media and social media. A dedicated webpage could be created to share communications, educational materials, and personal stories. Such narratives are powerful motivators for the public to seek sepsis-related information.

#### 3.1.4 Interventions to raise awareness among HCPs

Increasing awareness among HCPs primarily requires integrating education content into healthcare curricula. Most medical schools in Belgium have included sepsis in their curricula, yet no minimum formal learning objectives currently exist. Additionally, nursing programs need to strengthen their training in sepsis and infectious diseases. Undergraduate medical training should emphasize sepsis as a rare but specific condition.

A 2022 systematic review indicated that for nurses, interventions such as educational sessions, simulations, positive reinforcement through success stories in sepsis treatment, decision-support tools and standard protocols contribute to improved sepsis management (56). It is most likely that similar interventions would benefit healthcare providers working in long-term care facilities (LTCFs), although the available evidence for this group is limited. Implementing models for rapid structured communication are considered important for all healthcare providers (57, 58).

To ensure the effectiveness of these awareness-raising interventions among HCPs, it is essential to establish minimal requirements for training programs related to sepsis and to monitor the impact of educational interventions on HCP’s behavior and patient outcomes, through pre- and post-intervention assessments coupled with follow-up of patient outcomes.

### 3.2 Prevention of sepsis and safeguarding treatment options

When focusing on the prevention of sepsis, it is important to distinguish the different settings where sepsis may occur, as each setting comes with different challenges and possibilities. These settings include (1) the community (i.e., at home), (2) the acute healthcare setting (i.e., during or shortly after a hospitalization), and (3) the chronic care setting, i.e., LTCF. The acute healthcare setting is characterized by a high patient turnover and relative number of invasive procedures, but also by already existing infection prevention and control (IPC) activities and expertise.

#### 3.2.1 In the community

“Community-acquired sepsis” refers to sepsis complicating an infection or disease occurring at home. Given the heterogeneity of community-acquired sepsis, prevention needs to incorporate a broad set of actions. These include ensuring sufficient access to healthcare, improving health and vaccination literacy, enabling vaccination programs and offering adequate management of less severe infections with prudent use of antibiotics.

Access to healthcare is a crucial factor for preventing community-acquired sepsis and is influenced by a person’s social determinants of health. Key determinants relating to sepsis are socioeconomic status (i.e., person’s economic resources, education level, and occupation), access to healthcare services, housing conditions, administrative status, food safety, education level, occupational exposure, social networking and social status. The increase in non-communicable health and the overall aging of our population generated new, rapidly growing vulnerable groups. In addition, the growing number of patients with devices and catheters at home presents particular challenges. These patients, for instance, are more vulnerable to colonization with Methicillin-resistant *Staphylococcus aureus* (MRSA) and other multidrug-resistant organisms (MDROs), for which decolonization and follow-up programs need to be organized. Other societal phenomena, such as the increase of single-parent families and migration contribute to socioeconomic vulnerability. Homelessness is probably the most extreme social determinant jeopardizing health and a growing problem in several EU member states. Recent counting studies -by definition underestimated- mention at least 19.547 homeless persons in Flanders and at least 7,134 in Brussels (59). During the COVID-19 pandemic, homeless people experienced higher rates of hospitalization and mortality than the general population, lower vaccination rates, and suffered negative mental health impacts (60). Health literacy -the ability to access, understand, evaluate, and use health information to make informed decisions about health- is a critical factor for reducing community-acquired sepsis. Currently, one-third of the Belgian adult population has a low level of health literacy. Typically, people in poor health, older people, and lower educated people have a lower level of health literacy, although they have higher needs (61).

Vaccination programs for children and at-risk adults play a crucial role in preventing community-acquired sepsis. Vaccines for *Streptococcus pneumoniae*, *Neisseria meningitidis* and *Haemophiles influenzae*, but also for influenza and COVID-19, have proven effective in reducing the burden of pneumonia and meningitis potentially complicated by sepsis and death and even in reducing the burden of antimicrobial resistance. Children in Belgium receive these vaccines routinely in current vaccine programs, which has led to very high coverage rates in Belgium (62–64). However, for adults, most vaccines -except influenza and SARS-CoV2- are often costly and not systematically offered to high-risk persons. Moreover, people may not be aware of their high-risk status or lack confidence in or proper information about the required vaccinations. They might fear side effects or doubt the necessity or effectiveness. The Flemish regional government recently issued strategic targets for 2030 for adults, based on periodic assessments of vaccination coverage in target populations, which showed vaccination levels far below WHO targets. Specific targets for influenza vaccination include the ambition to reach 90% coverage in pregnant women, 80% in healthy persons ≥65 years old, and 90% in healthcare workers. For pneumococcal vaccination in healthy adults aged 65 and older, coverage rates should be at least 50% (65).

Preventing sepsis begins with awareness and early recognition, followed by appropriate management of infectious diseases regardless of their severity. This includes addressing urinary and respiratory tract infections as well as ensuring adequate wound and dental care. However, many infections are self-limiting and may not necessitate antibiotic treatment (66, 67). AMS programs aim to ensure appropriate antibiotic prescribing, balancing the need for timely infection treatment with efforts to avoid the overuse of antibiotics, which can lead to antimicrobial resistance (68). Particularly in primary care settings, diagnostic stewardship has emerged as a crucial aspect of AMS (69). Diagnostic stewardship aims to optimize the diagnostic process to ensure accurate identification of bacterial infections, thereby facilitating targeted antibiotic therapy while minimizing unnecessary antibiotic use.

#### 3.2.2 In the acute care (hospital) setting

Healthcare-associated infections (HAIs) are defined as infections that manifest during a patient’s hospitalization but are not yet present or incubating upon admission (70). Intrinsic risk factors for HAIs include patient-related factors such as age, immune status, co-morbidities, and acute conditions at the time of admission. Extrinsic factors involve endogenous sources, like previous colonization, and exogenous sources, such as contaminated hospital surfaces, equipment, or transmission via healthcare workers’ hands (71). Hospital-associated bloodstream infections are defined as laboratory-confirmed BSIs occurring two or more days after admission at the hospital. In Belgium, a 2022 report by Sciensano’s National Surveillance of Bloodstream Infections found a HABSI rate of 5.4 patients per 1,000 hospitalizations, with 43% of cases linked to invasive devices (34).

IPC interventions aim to reduce healthcare-associated sepsis and decrease the incidence of major types of preventable HAIs: central-line-associated BSIs, catheter-associated urinary tract infections (UTIs), surgical-site infections, *Clostridioides difficile* and hospital-acquired pneumonia (mainly ventilator-associated pneumonia) (72–74). The main IPC pillars are standard precautions, including hand hygiene, hygiene in the direct patient environment, use of personal protective equipment, sterilization of medical devices and waste management, transmission-based precautions, including adequate screening practices, and care bundles to prevent specific HAI or focused on specific pathogens (e.g., Vancomycin-Resistant *Enterococci*) (72). Overcrowding and inadequate staffing are important contributing factors to HAI, and this should also be acknowledged in any IPC planning.

Proper IPC planning demands adaptation to the local context, incorporating local and regional guidelines and programs. Actually available guidelines on IPC in Belgium include recommendations for hand hygiene, MDROs, BSIs related to intravascular catheters, UTIs, postoperative infections and the prevention of *Clostridium difficile* infections (75). However, guidelines for central-line associated BSI, for *Candida auris* infections and hospital- or ventilator-associated pneumonia prevention, as well as monitoring tools like compliance measurement systems, remain under development.

The adverse outcome of sepsis and the increasing challenge of AMR are inextricably linked, underscoring that any comprehensive program designed to improve outcomes in sepsis must incorporate AMS. Recent Surviving Sepsis Campaign guidelines address these concerns. National guidelines for antibiotic therapy, as issued by the BVIKM/SBIMC require urgent revision and should be easily available as a basis for empirical schemes in hospitals (76).

Numerous systematic reviews have investigated the influence of AMS interventions in hospital settings on individual patient outcomes, potentially mediated through sepsis. Generally, these interventions have demonstrated an overall reduction in antibiotic consumption without adverse effects on patients. Limited evidence suggests that specific AMS interventions, such as biomarker use, therapeutic drug monitoring and restrictions, are linked to improved outcomes, including reduced mortality and lower risk of superinfections (77). Evidence in pediatric settings and nursing homes is largely lacking (78).

In Belgian acute care hospitals, antibiotic policy groups or management teams are entrusted with facilitating AMS by developing antibiotic formularies and locally adapted guidelines for antibiotic use, providing permanent education and monitoring antibiotic consumption and resistance. These antibiotic management teams are positioned as a subgroup within their institution’s Drugs and Therapeutics Committee. Their composition, mandate and tasks are consolidated in the legislation on hospitals, and an annual budget of 3.6 million Euros is divided among these hospitals according to the number of beds (79–81).

#### 3.2.3 In the chronic care setting

LTCFs, such as nursing homes, institutions for persons with disabilities, rehabilitation centers, and psychiatric hospitals, provide home-replacing settings for persons with specific care needs because of age, frailty or physical or mental disabilities. Because of their context and population characteristics, the risk of HAI is high.

Since 2009, HAI and the use of antimicrobials in Belgian LTCF have been intermittently monitored as part of the ongoing HALT study (82). According to the 2021 report, the top 3 most reported HAIs are UTIs, respiratory infections and skin infections/gastro-intestinal infections. While all these can evolve into sepsis, the latter is currently not reported and thus unknown. Nursing homes are required to maintain a minimum record of specific data, but the structure for HAI surveillance in LTCFs is not standardized in Belgium. Participation in national surveillance programs is encouraged yet not mandatory. Moreover, the quality insurance of care in LTCFs is currently not a federal but a regional government responsibility.

Reducing HAI in LTCF by appropriate IPC interventions is clearly important. However, the available evidence on the effectiveness of these IPC interventions in LTCF is very limited. A systematic review by Lee et al. concluded that IPC programs, with at least four core elements from the WHO multimodal strategy, effectively reduced HAIs in nursing homes (72, 83). Enhancing hand hygiene, minimizing and improving catheter use, and employing enhanced barrier precautions, are practices that reduce UTIs in nursing home residents. However, most studies were underpowered to reach statistical significance (84). Wong et al. conducted a systematic review evaluating IPC interventions’ effectiveness in reducing MRSA. They could not find a clear benefit for specific interventions and thus concluded that standard precautions such as hand hygiene, environmental cleaning, and staff education, which are low-cost and not menacing to residents, should be the optimal approach in LTCFs (85).

In the effort to strengthen IPC in LTCFs, several initiatives have been launched in Belgium over the past years by either the federal or regional authorities, including two-yearly hand hygiene campaigns, a Flemish UTI prevention campaign, the new Flemish strategic vaccination objectives 2024–2030 and the specific incorporation of oral hygiene guidelines within the infection prevention instrument for nursing homes (65, 86–88).

A Belgian surveillance tool, the Infection Risk Scan (IRIS) (89), has become available to measure the quality of infection control and antimicrobial use and to support the implementation of specific improvement plans. Dedicated first-line outbreak support teams (whether or not in collaboration with hospital outbreak support teams) have started collaborations with LTCF to support such implementation practices.

Despite several recent investments in IPC strategies in Belgium, significant needs remain requiring focused action. In Belgium, LTCF-specific IPC guidelines are not yet available for many situations, commonly leading to the adaptation of hospital guidelines for use in LTCFs. The COVID-19 crisis in LTCFs has also revealed shortages of qualitative personal protective materials and sufficiently trained healthcare workers. Further on, the role and training of the coordinating LTCF clinician in IPC and AMS remains unclear.

Due to their possible frailty and gaps in guidelines, surveillance and HAI-trained staff, LTCF residents face increased risks from inappropriate antibiotic use. Symptoms of infection may be atypical, cognitive impairment limits communication of symptoms, and risks for the acquisition of resistant organisms are increased due to aging immune systems, complex comorbidities and frequent hospitalizations (90). Inappropriate antibiotic use results in increased risk of adverse drug events, *Clostridioides difficile* infection and infection with MDROs (91). The local coordinating physician could promote prudent antibiotic use and foster AMS in a LTCF. They currently have an unclear mandate and are often confronted with the prescription autonomy of colleagues (83, 84).

### 3.3 Early recognition and rapid response systems

Early recognition using standardized screening has a proven impact on outcomes in sepsis and is, as such, a key part of any sepsis quality improvement project (92, 93). Effective screening requires good test performance and integration into a system that ensures timely and adequate responses when specific thresholds are met. Notably, the test performance of such tools and the subsequent response certainly depends on the context in which it is implemented. In Belgium, evidence on the effectiveness of sepsis screening tools and rapid response systems across different care settings remains limited. Scheer et al.’s European survey suggests there is still significant room for improvement in sepsis recognition and response, particularly in standardizing screening protocols and ensuring adherence across healthcare facilities (94). Belgium could benefit from investing in tailored sepsis screening systems -such as the National Early Warning Score (NEWS)- that are adaptable to local contexts, combined with rapid response protocols to ensure early intervention and improved outcomes.

#### 3.3.1 Sepsis screening tools

##### 3.3.1.1 Primary care

Recognizing sepsis can be challenging in primary care due to lower *a priori* probability, delays in obtaining imaging or laboratory results, and other variables. Sepsis screening tools can assist primary care providers (PCPs) in recognizing sepsis and should be implemented as part of their standard care within a broader strategy, including patient education (95). Considering the local context and available resources, the National Early Warning Score (NEWS) might be most suitable, given its simplicity and the consistency with its use in other healthcare settings. Studies have found that higher NEWS values at GP referral are associated with faster medical review and poorer clinical outcomes in secondary care (96). NEWS has been successfully implemented in certain out-of-hours primary care services, showing continuous improvement in the proportion of patients with objective signs allowing NEWS calculation prior to hospital referral (97).

##### 3.3.1.2 Long-term facility care

For LTCF, solid evidence on screening tools with a sufficiently good test performance is still lacking. An early detection tool for sepsis in LTCF residents, developed by the Minnesota Hospital Association (3–100 s criteria) was described in a small observational study with a sensitivity of 79% and a specificity of 69% (98, 99). While evidence specific to LTCFs remains limited, studies suggest that NEWS, when adapted to the specific needs of frail older adult populations, can detect early clinical deterioration (100). Further validation in LTCF settings is required to confirm its utility and optimize thresholds to account for atypical presentations common in older adults.

##### 3.3.1.3 Emergency care

Emergency Departments (EDs) use triage systems, such as the Manchester triage system, to identify critically ill patients and provide thresholds and timings for subsequent medical consults and associated actions. Although not developed to do so, Manchester triage has shown acceptable sensitivity and negative predictive value for predicting sepsis in patients with fever (101, 102). The defined subsequent response is, however, not tailored to the needs of the sepsis patient, so additional screening and response tools are recommended. NEWS might provide added value when embedded in a rapid response system. Combining screening tools with early bedside point-of-care lactate testing is advised. Importantly, the performance requirements for cost-effective point-of-care sepsis tests in Belgium must still be explored (103). To further enhance sepsis recognition and response in EDs, advanced technologies such as machine learning offer significant potential (104).

##### 3.3.1.4 Intensive care unit ICU

Screening strategies for sepsis in the ICU are crucial for early detection and improved outcomes. Several scoring systems, including NEWS2 and biomarkers have been evaluated for their ability to predict sepsis and its severity. ICU settings present unique opportunities for developing and implementing machine learning and artificial intelligence to improve early sepsis detection. Several studies have demonstrated that machine learning models can enhance the predictive accuracy of early warning systems by analyzing vast amounts of real-time data, including vital signs, laboratory results, and clinical observations. For instance, machine learning models can identify subtle patterns of deterioration that may precede clinical recognition, leading to a reduction in sepsis-related mortality when integrated into routine ICU workflows (105, 106). Challenges remain, such as ensuring data interoperability, addressing ethical concerns related to AI decision-making, and validating these tools across diverse ICU populations.

##### 3.3.1.5 General ward

General ward care represents one of the most significant opportunities for improving sepsis outcomes, as the level of sepsis-specific knowledge and intensity of patient monitoring are often lower compared to critical care settings. Early recognition depends on standardized collection of vital signs and clinical observations, incorporated in clinical screening tools to allow for early supportive care. The NEWS has demonstrated strong performance in this setting, particularly when combined with a global “feeling of concern”—a clinical intuition frequently expressed by the bedside nurse. Nurse intuition can be integrated into NEWS adaptations or formalized by the Nurse Intuition Patient Deterioration Score (107, 108). In addition, continuous monitoring of vital signs using wearables or other smart devices is being investigated in both hospital wards and ambulatory care. Results remain yet conflicting regarding their clinical effectiveness, largely due to limitations in reliability, cost, and interoperability with existing hospital systems (109, 110).

##### 3.3.1.6 Screening in children and older adult

The performance of NEWS as an early warning tool is significantly lower in both children and older adult, necessitating the consideration of alternative scores (111).

In children, sepsis can present with atypical or nonspecific initial clinical symptoms. Although there is consensus about the benefits of early warning scores and rapid response teams, no existing score has demonstrated adequate test performance for this population. This partly stems from the lack of a pediatric-specific definition of sepsis (112–114). Recently, the Phoenix Sepsis Score, which provides international consensus criteria for pediatric sepsis, was published (115). This might provide a theoretical basis for the development of an updated pediatric early warning score.

Older adult patients with sepsis often present with atypical symptoms and signs. For example, older adult patients with sepsis may present with higher blood pressure because of arterial stiffness, a lower maximal heart rate, reduced arterial oxygen due to a ventilation-perfusion mismatch and lower body temperature. Considering these different physiological thresholds for clinical deterioration and changes in functional status, it is essential to validate and implement a specific EWS for older adult patients (116, 117).

#### 3.3.2 Implementing sepsis screening in Belgium

Any early warning score is only relevant if it is incorporated into a broader, well-coordinated system of early and appropriate response. Crossing specific score thresholds must trigger clear, standardized actions, such as intensified monitoring, timely bedside evaluation by trained providers, and escalation to advanced care when necessary. Without such a structured response system, their potential to improve patient outcomes and reduce delays in critical interventions is limited.

### 3.4 Early adequate treatment

Sepsis can lead to severe morbidity or mortality if not timely and appropriately treated. To describe factors that positively impact the outcome, we borrow two concepts well-known in the literature on sudden cardiac arrest. First, the Utstein Formula for Survival describes the potential for survival as the product of medical science, educational efficiency, and local implementation (118). Second, the Chain of Survival identifies all necessary steps to treat cardiac arrest (119). Suboptimal care in any given step will diminish the chances of good outcome, even if other steps are of high quality. This chain is equally applicable to sepsis, including the following linked parts: Early recognition and access to emergency medical care, early basic support, early advanced support, intensive care treatment, and recovery care. Optimizing care in each step should be based on scientific evidence but equally focused on education and implementation strategies (formula for Survival).

“Basic” sepsis care providers should be able to provide necessary early treatment, depending on their context and in line with their expected level of knowledge and skills. Basic sepsis care includes early fluid resuscitation, taking (blood) samples for biochemistry (serum lactate) and cultures, and starting appropriate antibiotics in a timely way (22, 120). Blood cultures remain the gold standard in the laboratory diagnosis of BSI and associated sepsis.

Healthcare systems should have dedicated procedures and always ensure easy access to support “basic” providers (121). Healthcare providers working with populations where sepsis presents atypical (e.g., young children and older adult), should be specifically trained to recognize sepsis and start early treatment. They should also be trained to prevent infection transmission. In addition, systems should have comprehensive “isolation” plans in place.

More “advanced” treatments should be the remit of “advanced care” teams, such as -for Belgium-the Prehospital Intervention Team (PIT), the physician-based “Mobiele Urgentiegroep” (MUG) or “Service Mobile d’Urgence et de Réanimation” (SMUR) teams, or the in-hospital rapid response teams. These dedicated teams should be specifically trained for the specific “advanced” medical interventions, for instance, the early initiation of vasoactive drug, and more broadly, for the “team-based” approach to care (112). They should institute further diagnostics to identify the focus of the underlying infection and sepsis-associated organ failure (22, 112, 122). The effectiveness of therapeutic interventions can be monitored in many ways, but should at least include monitoring of urine output, mean arterial blood pressure (MAP) and lactate clearance (123). Biomarkers might help diagnose sepsis. Despite some papers highlighting the potential of procalcitonin or pancreatic stone protein (PSP) (124), none of the more recent biomarkers has yet proven improved test performance compared to C-reactive protein (CRP) (22, 125). Finally, advanced care teams should have protocols and communication plans for the timely referral of critical patients to intensive care (22).

Adequate patient-centered intensive care is another important link in the chain of survival. Early rehabilitation is important, and the necessary staff should be available. Teams should have timely access to all diagnostic and therapeutic resources. Laboratories need to organize their processes so that preliminary and final results of blood cultures can be communicated as soon as possible. ICU and pediatric PICU should have specific procedures in place for the management of sepsis and septic shock patients, including adequate antibiotic management, early source control, advanced organ support, family-centered care and early rehabilitation. They should have a well-defined protocol for early referral of patients needing care that exceeds the capabilities of their department (22, 126–128).

Truly improving outcome demands not only a focus on science, but equally on education and implementation strategies. Providing education on sepsis and (early) treatment has proven to be a crucial yet challenging step (129, 130). Importantly, sepsis care is team-based, and a team approach should be an integral part of any training. Procedures should be in place to facilitate life-long learning rather than one-off initiatives (131, 132). Currently, no single optimal strategy is described in the literature to implement early adequate sepsis care. Combining educational programs and clinical-decision support tools is very effective (133, 134). Overcrowding at the emergency department and ICU can have a negative impact on guideline adherence (135, 136). Real-time electronic surveillance might help in such situations, yet studies fail to confirm this potential positive effect (137). Further identified barriers to guideline implementation are patient-related (advanced age, comorbidity, cryptic shock…) or organizational (clinician inexperience, lack of interprofessional collaboration, interhospital transfers, staff shortage) (121, 135, 138).

### 3.5 Post-sepsis care and rehabilitation

Post-Sepsis Syndrome (PSS) exists when there are consistent cognitive, emotional, physical, and medical defects after sepsis. PSS persists for weeks and even years after hospital discharge, occurring in more than half of sepsis survivors. In the worst cases, the sequelae can last for life. Patients hospitalized due to sepsis have an increased risk of readmission due to infection, cognitive impairment, mental and immunological problems, renal failure, and importantly cerebrovascular or cardiovascular events compared to non-sepsis hospitalized persons, as well as a higher risk of reduced quality of life (QoL) and death (24, 27, 139–143). Sepsis survivors are vulnerable to developing multi-organ and systemic sequelae and may experience exacerbation of existing medical conditions. Over half of them develop at least one new medical, psychological, or cognitive diagnosis after hospital discharge. Three out of ten will die during the first year after sepsis (26, 27, 141).

Physically, individuals may encounter challenges ranging from new functional limitations, muscle weakness and respiratory muscle weakness to amputations and cachexia (144–146). Critical illness polyneuropathy and myopathy can manifest alongside functional dependence and reduced endurance. The aftermath of sepsis often involves profound fatigue and dysphagia (147–149). Regarding neurocognitive impairments, dysfunctions were observed in attention and information processing, visuospatial or visuoperceptual, memory and executive functions (144, 150, 151). Mental health and overall well-being may be affected, with depression, anxiety, and posttraumatic stress symptoms impacting a notable portion of survivors (147). As the initial focus will be on organ failure and recovery, emotional and cognitive disorders in patients and/or family members might be diagnosed (too) late. Ideally, every sepsis patient (and caregivers) -as soon as he or she is cooperative- should, therefore, receive a brief screening in terms of cognition and mood. Post-sepsis sequelae also have an important economic impact due to both prolonged productivity loss and markedly increased long-term healthcare consumption (152–154).

There is a clear overlap between PSS and the recently described (conceptual broader) Post-Intensive Care Syndrome (PICS) (155). The PICS model describes how physical and neuropsychological impairments of the patient, and a decreased psychological well-being of family and caregivers can generate worse health-related QoL. Each of these pillars, in turn, then targets for therapeutic interventions (156).

By implementing an individualized, multidisciplinary approach, injury in sepsis can be mitigated and improve outcomes. Once hemodynamically stabilized, early nutritional support is crucial for patients with sepsis. Early mobilization is strongly advised between the second and fifth day after ICU admission (157). This proactive approach to rehabilitation can lead to various benefits, including improved ventilation, shortened ICU stays, reduced incidence of delirium or thromboembolism, increased walking distance, normalization of blood pressure and enhanced overall QoL. Mobilization can either be passive, for example by motorized devices, or active, once patient participation is possible (158).

Early occupational therapy further leads to reduced incidence and duration of delirium, decreased time on mechanical ventilation, shorter hospital stays, and cost savings (159, 160). Tasks include functional activities like active upper limb exercises, activities of daily living (ADL) training, and transfer training to maintain mobility and prevent complications like nerve injury and contractures (161). Occupational therapists also manage environmental controls, communication skills, and basic adaptations such as using adapted cutlery and ergonomic measures.

Being mechanically ventilated or experiencing severe critical illness are two major risk factors for dysphagia (162). Systematic screening for dysphagia is therefore recommended in all ICU (sepsis) patients. The diagnosis of dysphagia is made by a swallow evaluation conducted by a trained speech pathologist or by a flexible endoscopic evaluation of swallowing (148, 163). Possible treatment options for dysphagia include postural changes, dietary texture modifications, and interventions aiming to improve swallowing function (162).

Older adult patients are at increased risk of developing sepsis. The overall demand for ICU care in very old patients (80+) has increased significantly, even in those most frail. Properly determining the current health state and the frailty of such patients well before a potential ICU admission is important. This should lead to shared decision-making about the level of care wanted in certain circumstances (see 3.6 ethical considerations), taking into account the chances of survival with good functional outcome (145, 164–166). Children are equally at risk for long-term morbidity after sepsis. Compared with adults, children have a far greater range of developmental stages and therefore rehabilitation needs and goals, physical but also cognitive, emotional and (neuro)psychological (167, 168). Recent prospective data reported that up to 28% of children surviving sepsis developed a new disability by the time of hospital discharge (169). Neurophysiological and academic difficulties have also been described. Some families can demonstrate persistently elevated distress and family dysfunction. Psychological support and family education seem to play an even more vital role in long-term pediatric rehabilitation programs than in those for adults.

### 3.6 Ethical considerations

Sepsis is responsible for many deaths and might generate in those surviving long-term morbidity and associated economic costs (8, 165). Timely adequate prevention, recognition and treatment can improve outcomes. A Sepsis National Action Plan (SNAP) identifies strategies to do so. However, for all potential benefits, such a plan inherently incorporates risks. It can increase resource use and negatively impact proper AMS. It can create unrealistic expectations of outcomes and feed therapeutic tenacity (170).

The number of very old (80+) people in society is growing rapidly. When people over 80 are adequately informed about the impact and consequences of invasive procedures and treatment, such as invasive mechanical ventilation in the ICU, a significant proportion of them appear rather reluctant to accept life-sustaining treatments (171). Patients over 80 are, however, rarely asked for their opinion regarding a transfer to ICU (172). This entails the risk of over- or undertreatment. Treatment limitation decisions are often based on information collected in the first 48–72 h of ICU care, as individual patient’s values and preferences are not always clear upon admission. There is a clear risk for a self-fulfilling prophecy (173–177). Several studies show that the existence of for instance a do-not-attempt-resuscitation (DNAR) order, even if only for resuscitation, is in itself associated with decreased survival regardless of disease severity or comorbidities (178–182). While DNAR orders are not synonymous with “do not treat,” they may thus unintentionally limit aggressive treatment for, e.g., severe sepsis patients, especially in older adults.

In contrast, functional decline -even if substantial- does not necessarily lead to self-perceived poor QoL and likewise unwillingness to receive life-sustaining therapy (183–185). While many people in tempore non suspecto would identify a loss of independence as unacceptable, this may be far less clear once actual intensive care need occurs. This knowledge is important when guiding shared-decision making on ICU admission and life-sustaining treatments, taking into account that, even for frail patients, reliable risk prediction of long-term outcomes is sometimes difficult (176, 177, 185–189). Moreover, acutely occurring frailty might still be a dynamic condition and while it often worsens over time, it can also improve (190).

Considering the current ethical principles that guide our practice (autonomy, beneficence, nonmaleficence and justice), it is essential that an *a priori* discussion about goals of care should take place with the patient, his relatives and healthcare providers (Belgian Law Patient rights). Too often this only happens at the very end (if ever). A sepsis study in five US teaching hospitals (*n* = 2,956) indicated that patients who died during hospitalization had a reduced QoL even before hospitalization (191). They preferred less aggressive treatment, but less aggressive care was explicitly considered only when death was imminent. One out of four patients died with severe pain and one out of three with severe confusion. Advance care planning (ACP) based on shared decision-making might improve such reality (149, 189). It is important to understand that ACP differs from installing a DNAR order in many ways. Most DNAR orders are decided during hospitalization for severe conditions, most often in the first 24-48 h of admission (192). While it might include a specific DNAR order, ACP has and needs to have a much broader focus. It allows individuals to clearly define their goals and preferences for future care, to thoroughly discuss these with family and HCP, and to record and review these preferences as appropriate. ACP is ideally done before the actual deterioration occurs, although revision of the set goals might be considered at any moment.

Despite national guidance in many countries, the actual number of patients with available advance directives and/or defined care goals is limited, even in those with a high risk of adverse outcomes (186, 193–198). For instance, in Flemish residential care centers, where the oldest and most vulnerable patients reside, 40% did not have an ACP in 2022 (199).

For many patients, palliative care might be appropriate as part of their ACP. Palliative care focuses on the relief of suffering, but not necessarily limits any further treatment of for instance comorbidities (165). It is not because people are in palliative care that they would not call upon emergency services, nor that they would not deserve treatment under certain conditions. Many would not want additional harm from hospitalization, but would still profit from short-term therapies, such as intravenous antibiotics or oxygen, if these could be provided in their own environment (200–203). Many healthcare providers find it challenging to withdraw a therapy initiated in an emergency, even when it is no longer in the patient’s best interest. However, from an ethical perspective, discontinuing such therapy may often be the more appropriate choice, particularly when done in a comfortable and controlled manner. Healthcare providers should be aware of potential barriers to appropriate palliative care and try to improve them. For instance, patients and families might disagree among themselves. Healthcare providers might have difficulty estimating their patient’s decision-making capacity. Considering that ACP is time and resource-consuming, there is also a need for additional incentives, either legal or financial.

ACP should be an integral part of any large-scale communication about critical illness and, specifically, sepsis. Finally, to prevent miscommunication or ambiguity and avoid delay in appropriate care, it is important that a patient’s advance directives become available and accessible at any moment 24/7 for all involved healthcare providers, via for instance an electronic platform, the patient’s e-health box, passport or other.

### 3.7 Surveillance and research

Our understanding of the incidence, circumstances, care provided, and outcomes for sepsis patients within the Belgian healthcare system needs to be completed. Current surveillance efforts -by Sciensano, the federal government of health research institute- primarily concentrate on hospital-acquired infections, such as HABSI (34). This registry is a mandatory annual surveillance regulated by Royal Decree. In addition, the European point prevalence study on HAI and antimicrobial use provides a comprehensive EU-wide perspective, capturing data from about 40% of Belgian general hospitals every 5 years (204). However, these surveillance systems have significant gaps and limitations. They do not specifically collect data on sepsis and septic shock as defined in the Sepsis-3 definitions. Additionally, there is a problematic delay in the reporting of this information.

The Monitoring Intensive Care Activities (MICA) project, which gathers data directly from Patient Data Management Systems (PDMS) in ICUs in selected hospitals, could potentially serve as a valuable resource for sepsis data (205). The reliability of sepsis data coding, such as ICD9 and ICD10, is questionable, adding another layer of complexity to the accurate tracking and analysis of sepsis within the country.

A collaborative effort to refine data collection and reporting mechanisms is urgently needed to address these issues and improve our understanding of the epidemiology and management of sepsis. The objectives of this sepsis surveillance should include (1) monitoring the evolution of sepsis and septic shock prevalence and incidence, and thereby assessing the impact of the SNAP with its proposed interventions, (2) estimating the burden of disease caused by sepsis/septic shock and (3) assessing the implementation of the different components of the SNAP in the community and first-line healthcare, hospitals and LTCFs.

Research is indispensable in the ongoing effort to advance the quality of care for sepsis patients in Belgium. Funding is available for sepsis research, including grants that focus on international collaboration. However, a notable challenge is the categorization of sepsis within funding schemes. Sepsis research often competes for attention and funding resources in broader categories, such as basic research and immunology, rather than being recognized as an individual topic. Despite the extensive research efforts on sepsis in Belgium, there is a lack of coordination and collaboration among hospitals and institutions. This fragmentation hinders progress in understanding and treating sepsis, as more cohesive efforts could lead to significant advancements.

## 4 Actionable recommendations

Overview of actionable recommendations is detailed in Supplementary material.

## 5 Discussion

Sepsis is a major healthcare problem leading to death or prolonged morbidity for both patients and their caregivers. In addition, it generates a significant burden on healthcare systems and society. Unsurprisingly, the WHA identified sepsis as a health priority and urged member states to develop and implement national strategies for sepsis prevention, diagnosis, and management.

Evidence from other countries or regions demonstrates that coordinated programs in collaboration with governments, professionals and patient-advocacy groups can save lives, improve the outcomes for sepsis survivors, and reduce costs for the healthcare system. A SNAP outlines a comprehensive, multidisciplinary, multi-stakeholder approach to prevent or mitigate the burden of sepsis. This approach includes the coordinated efforts of the various stakeholders, the necessary resources, and the political decisions that need to be made. It provides a roadmap and structured framework for implementing and monitoring essential interventions, ensuring synergy with other existing programs.

The Belgian Sepsis National Action Plan (Be-SNAP) initiative is the collaborative effort of many, including but not limited to the major scientific societies involved, patient representatives and policy makers. Drawing on theoretical frameworks and the opinions and perspectives of each of the mentioned actors, seven priority topics were identified and further examined within the context of the Belgian healthcare system. Such an exploration is limited by the lack of Belgian data and previous coordination and that already constituted some of the recommendations the SNAP initiative eventually proposed.

While we aimed to evaluate each priority topic for its impact, its feasibility and the anticipated cost-effectiveness, we identified some blind spots in our evaluation, particularly in predicting potential risks associated with the implementation of the SNAP such as resource overuse and antibiotic misuse. For example, setting up a national campaign to raise awareness among the general public might lead to increased concern with minor illness episodes and increased use of first line health care services, with a risk for increased demand for antimicrobial use for less severe febrile illnesses. Likewise, enhanced attention for sepsis screening at emergency departments might have unintended perverse effects of stimulating overuse of broad spectrum antimicrobials. As both phenomena are strongly dependent on perceptions, beliefs and behavior, the real world impact in the Belgian health care system is difficult to estimate. To address these challenges, it is crucial to proceed in a coordinated and structured manner. Adopting a continuous improvement methodology—such as the Plan-Do-Check-Act (PDCA) cycle—can help systematically monitor, evaluate, and mitigate potential unintended consequences at each stage of the rollout.

Effective implementation will require close collaboration with existing healthcare structures and actors. Policymakers need to be well-informed and actively involved from the setup and implementation phases, as many of the recommendations require either financial or legislation support. Alongside policy makers, a detailed roadmap for the SNAP implementation should be created. The initial focus of such a roadmap should be twofold: first, establishing the overarching necessary structures, and second, identifying interventions that offer a better balance of cost efficiency and ease of implementation. Other interventions can be gradually introduced within the existing SNAP structure.

A pivotal recommendation involves the creation of the National Sepsis Forum and Foundation, which would serve as central coordinators and advocates for sepsis-related initiatives. These organizations could ensure alignment across stakeholders, promote best practices, and drive quality improvement efforts. Moreover, ongoing monitoring, combined with periodic evaluations, will allow for the identification of challenges and successes, enabling evidence-driven adaptations to the program.

Future iterations of this initiative should prioritize an even broader inclusion of stakeholders, including public health officials, community health organizations and local health authorities. Incorporating input from representatives of governmental health agencies and policymakers is essential to align the action plan with national health priorities and funding strategies. Collaborating with academic institutions for research support could facilitate the integration of the latest scientific findings into practice. Furthermore, involving industry stakeholders would foster dialog around innovations in diagnostics and treatment options for sepsis. Lastly, engaging with health insurance providers could ensure that the financial aspects of sepsis management are adequately addressed, promoting equitable access to care. By encompassing this wider array of stakeholders, the action plan can be more comprehensive, addressing the multifaceted challenges of sepsis management more effectively.

To conclude, we want to highlight three key areas we believe have the greatest potential for impact and cost-effectiveness. First, we need to improve early recognition of sepsis to ensure timely and adequate treatment. This improvement requires the implementation of validated early warning systems tailored to specific settings. Training healthcare providers to effectively use these tools, combined with the integration of technology such as point-of-care testing and digital monitoring systems, will further enhance early detection.

Second, while all stakeholders have a role to play, the success of these efforts fundamentally depends on strong government support. Financial investment, targeted legislation, and regulatory frameworks are necessary to enable implementation, scale interventions, and ensure sustainability. A formalized national sepsis strategy, supported by government-backed incentives, could align stakeholders and provide a clear roadmap for coordinated action.

Lastly, in view of long-term effectiveness and continuous improvement, the mandatory registration and monitoring of Belgian sepsis data is crucial. Establishing a centralized Belgian Sepsis Registry will provide valuable insights into the epidemiology, outcomes, and quality of sepsis care, facilitating evidence-based decision-making and benchmarking against international standards. This registry can drive research, highlight gaps in care, and monitor the impact of newly implemented interventions.

By fostering collaboration, building robust structures, and prioritizing evidence-based implementation, the SNAP initiative has the potential to transform sepsis care in Belgium, ensuring better patient outcomes, resource optimization, and a healthcare system that is both resilient and prepared to address this critical challenge. Furthermore, this structured approach can serve as an inspiring model for other countries facing similar challenges, highlighting one of the key aims of this work: to drive meaningful change both nationally and internationally in the fight against sepsis.

## References

[1] Reinhart K Daniels R Kissoon N Machado FR Schachter RD Finfer S. Recognizing Sepsis as a Global Health priority - a WHO resolution. N Engl J Med. (2017) 377:414–7. doi: 10.1056/NEJMp1707170, PMID: 28658587

[2] World Health Assembly. WHA adopts resolution on sepsis (2017). Available online at: https://www.global-sepsis-alliance.org/news/2017/5/26/wha-adopts-resolution-on-sepsis (Accessed February 2, 2025).

[3] Hotchkiss RS Moldawer LL Opal SM Reinhart K Turnbull IR Vincent JL. Sepsis and septic shock. Nat Rev Dis Primers. (2016) 2:16045. doi: 10.1038/nrdp.2016.45, PMID: 28117397 PMC5538252

[4] Singer M Deutschman CS Seymour CW Shankar-Hari M Annane D Bauer M. The third international consensus definitions for Sepsis and septic shock (Sepsis-3). JAMA. (2016) 315:801–10. doi: 10.1001/jama.2016.0287, PMID: 26903338 PMC4968574

[5] World Health Organization. Fact sheet sepsis (2024). Available online at: https://www.who.int/news-room/fact-sheets/detail/sepsis (Accessed February 2, 2025).

[6] Cecconi M Evans L Levy M Rhodes A. Sepsis and septic shock. Lancet. (2018) 392:75–87. doi: 10.1016/S0140-6736(18)30696-2, PMID: 29937192

[7] Nasir N Jamil B Siddiqui S Talat N Khan FA Hussain R. Mortality in Sepsis and its relationship with gender. Pak J Med Sci. (2015) 31:1201–6. doi: 10.12669/pjms.315.6925, PMID: 26649014 PMC4641283

[8] Stenberg H Li X Pello-Esso W Larsson Lonn S Thonnings S Khoshnood A. The effects of sociodemographic factors and comorbidities on sepsis: a nationwide Swedish cohort study. Prev Med Rep. (2023) 35:102326. doi: 10.1016/j.pmedr.2023.102326, PMID: 37519448 PMC10374593

[9] Plaeke P De Man JG Coenen S Jorens PG De Winter BY Hubens G. Clinical- and surgery-specific risk factors for post-operative sepsis: a systematic review and meta-analysis of over 30 million patients. Surg Today. (2020) 50:427–39. doi: 10.1007/s00595-019-01827-4, PMID: 31172283

[10] Bonet M Souza JP Abalos E Fawole B Knight M Kouanda S. The global maternal sepsis study and awareness campaign (GLOSS): study protocol. Reprod Health. (2018) 15:16. doi: 10.1186/s12978-017-0437-8, PMID: 29382352 PMC5791346

[11] Moore JX Akinyemiju T Bartolucci A Wang HE Waterbor J Griffin R. A prospective study of cancer survivors and risk of sepsis within the REGARDS cohort. Cancer Epidemiol. (2018) 55:30–8. doi: 10.1016/j.canep.2018.05.001, PMID: 29763753 PMC6054880

[12] Liu Q Song H Andersson TM Magnusson PKE Zhu J Smedby KE. Psychiatric disorders are associated with increased risk of sepsis following a cancer diagnosis. Cancer Res. (2020) 80:3436–42. doi: 10.1158/0008-5472.CAN-20-0502, PMID: 32532824

[13] Estenssoro E Loudet CI Edul VSK Osatnik J Rios FG Vasquez DN. Health inequities in the diagnosis and outcome of sepsis in Argentina: a prospective cohort study. Crit Care. (2019) 23:250. doi: 10.1186/s13054-019-2522-6, PMID: 31288865 PMC6615149

[14] Born S Fleischmann-Struzek C Abels W Piedmont S Neugebauer E Reinhart K. Most patients with an increased risk for sepsis-related morbidity or death do not recognize sepsis as a medical emergency: results of a survey study using case vignettes. Crit Care. (2023) 27:446. doi: 10.1186/s13054-023-04733-x, PMID: 37978408 PMC10655489

[15] Shankar-Hari M Saha R Wilson J Prescott HC Harrison D Rowan K. Rate and risk factors for rehospitalisation in sepsis survivors: systematic review and meta-analysis. Intensive Care Med. (2020) 46:619–36. doi: 10.1007/s00134-019-05908-3, PMID: 31974919 PMC7222906

[16] Galiatsatos P Sun J Welsh J Suffredini A. Health disparities and Sepsis: a systematic review and Meta-analysis on the influence of race on Sepsis-related mortality. J Racial Ethn Health Disparities. (2019) 6:900–8. doi: 10.1007/s40615-019-00590-z, PMID: 31144133 PMC10875732

[17] Linnander EL Ayedun A Boatright D Ackerman-Barger K Morgenthaler TI Ray N. Mitigating structural racism to reduce inequities in sepsis outcomes: a mixed methods, longitudinal intervention study. BMC Health Serv Res. (2022) 22:975. doi: 10.1186/s12913-022-08331-5, PMID: 35907839 PMC9338573

[18] Lin GL McGinley JP Drysdale SB Pollard AJ. Epidemiology and immune pathogenesis of viral sepsis. Front Immunol. (2018) 9:2147. doi: 10.3389/fimmu.2018.02147 30319615 PMC6170629

[19] Society of Critical Care Medicine. Surviving Sepsis campaign (2024). Available online at: https://www.sccm.org/survivingsepsiscampaign (Accessed February 2, 2025).

[20] Nannan Panday RS Lammers EMJ Alam N Nanayakkara PWB. An overview of positive cultures and clinical outcomes in septic patients: a sub-analysis of the prehospital antibiotics against Sepsis (PHANTASi) trial. Crit Care. (2019) 23:182. doi: 10.1186/s13054-019-2431-8, PMID: 31113475 PMC6530106

[21] Loots FJ Smits M van Steensel C Giesen P Hopstaken RM van Zanten ARH. Management of sepsis in out-of-hours primary care: a retrospective study of patients admitted to the intensive care unit. BMJ Open. (2018) 8:e022832. doi: 10.1136/bmjopen-2018-022832, PMID: 30224394 PMC6144400

[22] Evans L Rhodes A Alhazzani W Antonelli M Coopersmith CM French C. Surviving sepsis campaign: international guidelines for management of sepsis and septic shock 2021. Intensive Care Med. (2021) 47:1181–247. doi: 10.1007/s00134-021-06506-y, PMID: 34599691 PMC8486643

[23] GBD 2021 Antimicrobial Resistance Collaborators. Global burden of bacterial antimicrobial resistance 1990-2021: a systematic analysis with forecasts to 2050. Lancet. (2024) 404:1199–226. doi: 10.1016/S0140-6736(24)01867-1 39299261 PMC11718157

[24] Kosyakovsky LB Angriman F Katz E Adhikari NK Godoy LC Marshall JC. Association between sepsis survivorship and long-term cardiovascular outcomes in adults: a systematic review and meta-analysis. Intensive Care Med. (2021) 47:931–42. doi: 10.1007/s00134-021-06479-y, PMID: 34373953

[25] McNamara JF Righi E Wright H Hartel GF Harris PNA Paterson DL. Long-term morbidity and mortality following bloodstream infection: a systematic literature review. J Infect. (2018) 77:1–8. doi: 10.1016/j.jinf.2018.03.005, PMID: 29746948

[26] Spoden M Hartog CS Schlattmann P Freytag A Ostermann M Wedekind L. Occurrence and risk factors for new dependency on chronic care, respiratory support, Dialysis and mortality in the first year after Sepsis. Front Med (Lausanne). (2022) 9:878337. doi: 10.3389/fmed.2022.878337, PMID: 35665356 PMC9162443

[27] Fleischmann-Struzek C Rose N Freytag A Spoden M Prescott HC Schettler A. Epidemiology and costs of Postsepsis morbidity, nursing care dependency, and mortality in Germany, 2013 to 2017. JAMA Netw Open. (2021) 4:e2134290. doi: 10.1001/jamanetworkopen.2021.34290, PMID: 34767025 PMC8590172

[28] World Health Organization. Global report on the epidemiology and burden of sepsis (2020). https://iris.who.int/bitstream/handle/10665/334216/9789240010789-eng.pdf?sequence=1 (Accessed February 2, 2025).

[29] Cohen J Vincent JL Adhikari NK Machado FR Angus DC Calandra T. Sepsis: a roadmap for future research. Lancet Infect Dis. (2015) 15:581–614. doi: 10.1016/S1473-3099(15)70112-X, PMID: 25932591

[30] Rudd KE Kissoon N Limmathurotsakul D Bory S Mutahunga B Seymour CW. The global burden of sepsis: barriers and potential solutions. Crit Care. (2018) 22:232. doi: 10.1186/s13054-018-2157-z, PMID: 30243300 PMC6151187

[31] Daviaud F Grimaldi D Dechartres A Charpentier J Geri G Marin N. Timing and causes of death in septic shock. Ann Intensive Care. (2015) 5:16. doi: 10.1186/s13613-015-0058-8, PMID: 26092499 PMC4474967

[32] D'Onofrio V Meersman A Vijgen S Cartuyvels R Messiaen P Gyssens IC. Risk factors for mortality, intensive care unit admission, and bacteremia in patients suspected of sepsis at the emergency department: a prospective cohort study. Open Forum Infect Dis. (2021) 8:ofaa594. doi: 10.1093/ofid/ofaa594, PMID: 33511231 PMC7813192

[33] Rudd KE Johnson SC Agesa KM Shackelford KA Tsoi D Kievlan DR. Global, regional, and national sepsis incidence and mortality, 1990-2017: analysis for the global burden of disease study. Lancet. (2020) 395:200–11. doi: 10.1016/S0140-6736(19)32989-7, PMID: 31954465 PMC6970225

[34] Sciensano. (2023). Surveillance of bloodstream infections in Belgian hospitals annual report (2023). Available online at: https://www.sciensano.be/sites/default/files/sciensano_national_bloodstream_infection_report_2023_1.pdf (Accessed February 2, 2025).

[35] Van de Voorde P Monsieurs KG Perkins GD Castren M. Looking over the wall: using a Haddon matrix to guide public policy making on the problem of sudden cardiac arrest. Resuscitation. (2014) 85:602–5. doi: 10.1016/j.resuscitation.2014.01.032, PMID: 24530250

[36] Haddon W Jr. Advances in the epidemiology of injuries as a basis for public policy. Public Health Rep. (1980) 95:411–21. 7422807 PMC1422748

[37] Barnett DJ Balicer RD Blodgett D Fews AL Parker CL Links JM. The application of the Haddon matrix to public health readiness and response planning. Environ Health Perspect. (2005) 113:561–6. doi: 10.1289/ehp.7491, PMID: 15866764 PMC1257548

[38] Lett R Kobusingye O Sethi D. A unified framework for injury control: the public health approach and Haddon's matrix combined. Inj Control Saf Promot. (2002) 9:199–205. doi: 10.1076/icsp.9.3.199.8708, PMID: 12462174

[39] Runyan CW. Using the Haddon matrix: introducing the third dimension. Inj Prev. (1998) 4:302–7. doi: 10.1136/ip.4.4.302, PMID: 9887425 PMC1730420

[40] Public Health England. (2019). Achieving behaviour change: a guide for local government and partners. https://assets.publishing.service.gov.uk/media/5e7b4e85d3bf7f133c923435/PHEBI_Achieving_Behaviour_Change_Local_Government.pdf (Accessed February 2, 2025).

[41] Smela B Toumi M Świerk K Francois C Biernikiewicz M Clay E. Rapid literature review: definition and methodology. J Mark Access Health Policy. (2023) 11:2241234. doi: 10.1080/20016689.2023.2241234, PMID: 37533549 PMC10392303

[42] Kahan DM. Social science. A risky science communication environment for vaccines. Science. (2013) 342:53–4. doi: 10.1126/science.1245724, PMID: 24092722

[43] Sepsibel. Onderzoek iVox: maar 1 op de 5 Belgen weet wat sepsis is (2024). Available online at: https://www.sepsibel.be/algemeen/onderzoek-ivox-maar-1-op-de-5-belgen-weet-wat-sepsis-is/ (Accessed February 2, 2025).

[44] Fiest KM Krewulak KD Brundin-Mather R Leia MP Fox-Robichaud A Lamontagne F. Patient, public, and healthcare professionals' Sepsis awareness, knowledge, and information seeking behaviors: a scoping review. Crit Care Med. (2022) 50:1187–97. doi: 10.1097/CCM.0000000000005564, PMID: 35481953 PMC9275848

[45] Medeiros DNM Shibata AO Pizarro CF Rosa MLA Cardoso MP Troster EJ. Barriers and proposed solutions to a successful implementation of pediatric sepsis protocols. Front Pediatr. (2021) 9:755484. doi: 10.3389/fped.2021.755484 34858905 PMC8631453

[46] de Souza DC Barreira ER Faria LS. The epidemiology of Sepsis in childhood. Shock. (2017) 47:2–5. doi: 10.1097/SHK.0000000000000699, PMID: 27454387

[47] Peters E Rhodes A Measey MA Babl FE Long E. Sepsis awareness and understanding in Australian parents: a National Child Health Poll survey. J Paediatr Child Health. (2023) 59:1047–52. doi: 10.1111/jpc.16453, PMID: 37326211

[48] Mulders MCF Loots FJ van Nieuwenhoven J Ter Maaten JC Bouma HR. Use of sepsis-related diagnostic criteria in primary care: a survey among general practitioners. Fam Pract. (2021) 38:617–22. doi: 10.1093/fampra/cmab020, PMID: 33755106 PMC8527837

[49] Atkins PE Bastin MLT Morgan RJ Laine ME Flannery AH. Pharmacist involvement in sepsis response and time to antibiotics: a systematic review. J Am Coll Clin Pharm. (2023) 6:942–53. doi: 10.1002/jac5.1723, PMID: 37608990 PMC10441617

[50] Eitze S Fleischmann-Struzek C Betsch C Reinhart K. Determinants of sepsis knowledge: a representative survey of the elderly population in Germany. Crit Care. (2018) 22:273. doi: 10.1186/s13054-018-2208-5, PMID: 30368239 PMC6204268

[51] Mellhammar L Christensson B Linder A. Public awareness of sepsis is low in Sweden. Open Forum Infect Dis. (2015) 2:ofv161. doi: 10.1093/ofid/ofv161 26634220 PMC4664835

[52] Bray JE Johnson R Trobbiani K Mosley I Lalor E Cadilhac D. Australian public's awareness of stroke warning signs improves after national multimedia campaigns. Stroke. (2013) 44:3540–3. doi: 10.1161/STROKEAHA.113.002987, PMID: 24135926

[53] Rush L Hilton S McDaid L. A simple dose of antibiotics: qualitative analysis of sepsis reporting in UK newspapers. BJGP Open. (2020) 4:bjgpopen20X101005. doi: 10.3399/bjgpopen20X101005, PMID: 31964635 PMC7330194

[54] Gaspoz JM Unger PF Urban P Chevrolet JC Rutishauser W Lovis C. Impact of a public campaign on pre-hospital delay in patients reporting chest pain. Heart. (1996) 76:150–5. doi: 10.1136/hrt.76.2.150, PMID: 8795479 PMC484464

[55] NICE. NICE guideline [NG143]: Fever in under 5s: Assessment and initial management. (2021). Available online at: https://www.nice.org.uk/guidance/ng143 (Accessed February 2, 2025).

[56] Rababa M Bani Hamad D Hayajneh AA. Sepsis assessment and management in critically ill adults: a systematic review. PLoS One. (2022) 17:e0270711. doi: 10.1371/journal.pone.0270711, PMID: 35776738 PMC9249173

[57] Mihaljevic SE Howard VM. Incorporating Interprofessional evidenced-based Sepsis simulation education for certified nursing assistants (CNAs) and licensed care providers within Long-term care settings for process and quality improvement. Crit Care Nurs Q. (2016) 39:24–33. doi: 10.1097/CNQ.0000000000000092, PMID: 26633155

[58] Ahmed AM Macapili E Brenner MJ Pandian V. Accelerating detection and intervention for Sepsis in skilled nursing facilities using a Sepsis pathway. J Nurs Care Qual. (2024) 39:67–75. doi: 10.1097/NCQ.0000000000000729, PMID: 37350588

[59] LUCAS KU Leuven. Tellingen dak- en thuisloosheid Globaal Rapport 2023 (2023). Available online at: https://www.vlaanderen.be/gezondheid-en-welzijn/armoede/armoedebestrijding-in-vlaanderen/tellingen-dak-en-thuisloosheid/tellingen-dak-en-thuisloosheid-2023 (Accessed February 2, 2025).

[60] Mackey K Ayers CK Kondo KK Saha S Advani SM Young S. Racial and ethnic disparities in COVID-19-related infections, hospitalizations, and deaths: a systematic review. Ann Intern Med. (2021) 174:362–73. doi: 10.7326/M20-6306, PMID: 33253040 PMC7772883

[61] Sciensano. Determinants of health: Health literacy, health status report (2020). Available online at: https://www.healthybelgium.be/en/health-status/determinants-of-health/health-literacy (Accessed February 2, 2025).

[62] Hoge Gezondheidsraad. Basisvaccinatie (2021). Available online at: https://www.hgr-css.be/nl/advies/9606/basisvaccinatieschema (Accessed February 2, 2025).

[63] Hoge Gezondheidsraad. Vaccinatie van kinderen en adolescenten tegen pneumokokken. (2023). Available online at: https://www.laatjevaccineren.be/sites/default/files/2024-02/20230117_hgr-9746_vaccinatie_tegen_pneumokokken_vweb.pdf (Accessed February 2, 2025).

[64] Hoge Gezondheidsraad. Vaccination of immunocompromised or chronically ill children and/or adults (2019). Available online at: https://www.hgr-css.be/file/download/f0929f16-6ab7-4ba7-988f-941b69d5de01/L572yvPPRtKJIqwkOSAZXbBx5eaSeRv8hORKFCPso3d.pdf (Accessed February 2, 2025).

[65] Vlaamse Raad Welzijn Volksgezondheid Gezin. Advies Gezondheidsdoelstelling Vaccinatie (2024). Available online at: https://laatjevaccineren.paddlecms.net/sites/default/files/2024-02/18_01_GC_Vaccinaties_Voorbereidend_rapport_2024.pdf (Accessed February 2, 2025).

[66] Malhotra-Kumar S Lammens C Coenen S Van Herck K Goossens H. Effect of azithromycin and clarithromycin therapy on pharyngeal carriage of macrolide-resistant streptococci in healthy volunteers: a randomised, double-blind, placebo-controlled study. Lancet. (2007) 369:482–90. doi: 10.1016/S0140-6736(07)60235-9, PMID: 17292768

[67] Malhotra-Kumar S Van Heirstraeten L Coenen S Lammens C Adriaenssens N Kowalczyk A. Impact of amoxicillin therapy on resistance selection in patients with community-acquired lower respiratory tract infections: a randomized, placebo-controlled study. J Antimicrob Chemother. (2016) 71:3258–67. doi: 10.1093/jac/dkw234, PMID: 27353466

[68] Dyar OJ Huttner B Schouten J Pulcini C. What is antimicrobial stewardship? Clin Microbiol Infect. (2017) 23:793–8. doi: 10.1016/j.cmi.2017.08.026, PMID: 28882725

[69] Bruyndonckx R Adriaenssens N Versporten A Hens N Monnet DL Molenberghs G. Consumption of antibiotics in the community, European Union/European economic area, 1997-2017. J Antimicrob Chemother. (2021) 76:ii7–ii13. 34312654 10.1093/jac/dkab172 PMC8314117

[70] Centers for Disease Control and Prevention. About healthcare-associated infections (2024). Available online at: https://www.cdc.gov/healthcare-associated-infections/about/index.html (Accessed February 2, 2025).

[71] Rodríguez-Acelas AL de Abreu Almeida M Engelman B Cañon-Montañez W. Risk factors for health care-associated infection in hospitalized adults: systematic review and meta-analysis. Am J Infect Control. (2017) 45:e149–56. doi: 10.1016/j.ajic.2017.08.016 29031433

[72] World Health Organization. Guidelines on core components of infection prevention and control programmes at the national and acute health care facility level. (2016). Available online at: https://www.who.int/publications/i/item/9789241549929 (Accessed February 3, 2025). 27977095

[73] Yokoe DS Advani SD Anderson DJ Babcock HM Bell M Berenholtz SM. Introduction to a compendium of strategies to prevent healthcare-associated infections in acute-care hospitals: 2022 updates. Infect Control Hosp Epidemiol. (2023) 44:1533–9. doi: 10.1017/ice.2023.158, PMID: 37855077 PMC10587365

[74] Trivedi KK Schaffzin JK Deloney VM Aureden K Carrico R Garcia-Houchins S. Implementing strategies to prevent infections in acute-care settings. Infect Control Hosp Epidemiol. (2023) 44:1232–46. doi: 10.1017/ice.2023.103, PMID: 37431239 PMC10527889

[75] Hoge Gezondheidsraad. AANBEVELINGEN INZAKE PREVENTIE, BEHEERSING EN AANPAK VAN PATIËNTEN DIE DRAGER ZIJN VAN TEGEN ANTIBIOTICA MULTIRESISTENTE ORGANISMEN (MDRO) IN ZORGINSTELLINGEN (2019). Available online at: https://www.health.belgium.be/sites/default/files/uploads/fields/fpshealth_theme_file/20221130_hgr-9277_mdro_update_vweb_1.pdf (Accessed February 2, 2025).

[76] Belgische Vereniging voor Infectiologie en Klinische Microbiologie (2019). Available online at: https://www.bvikm.org/iggi-info-nl (Accessed February 2, 2025).

[77] Schuts EC Hulscher M Mouton JW Verduin CM Stuart J Overdiek H. Current evidence on hospital antimicrobial stewardship objectives: a systematic review and meta-analysis. Lancet Infect Dis. (2016) 16:847–56. doi: 10.1016/S1473-3099(16)00065-7, PMID: 26947617

[78] D'Onofrio V Meersman A Magerman K Waumans L van Halem K Cox JA. Audit of empirical antibiotic therapy for sepsis and the impact of early multidisciplinary consultation on patient outcomes. Int J Antimicrob Agents. (2021) 58:106379. doi: 10.1016/j.ijantimicag.2021.106379, PMID: 34161787

[79] Sourdeau L Struelens MJ Peetermans WE Costers M Suetens C. Implementation of antibiotic management teams in Belgian hospitals. Acta Clin Belg. (2006) 61:58–63. doi: 10.1179/acb.2006.011, PMID: 16792335

[80] Van Gastel E Costers M Peetermans WE Struelens MJ. Nationwide implementation of antibiotic management teams in Belgian hospitals: a self-reporting survey. J Antimicrob Chemother. (2010) 65:576–80. doi: 10.1093/jac/dkp470, PMID: 20053695

[81] Van Gastel E Balligand E Costers M Magerman K. Antibiotic management teams in Belgian hospitals: continued improvement in the period from 2007 to 2011. Eur J Clin Microbiol Infect Dis. (2015) 34:673–7. doi: 10.1007/s10096-014-2279-4, PMID: 25407373

[82] Sciensano. NSIH-LTCF - healthcare-associated infections in long-term care facilities HALT (2021). Available online at: https://www.sciensano.be/en/projects/healthcare-associated-infections-long-term-care-facilities (Accessed February 2, 2025).

[83] Lee MH Lee GA Lee SH Park YH. Effectiveness and core components of infection prevention and control programmes in long-term care facilities: a systematic review. J Hosp Infect. (2019) 102:377–93. doi: 10.1016/j.jhin.2019.02.008, PMID: 30794854

[84] Meddings J Saint S Krein SL Gaies E Reichert H Hickner A. Systematic review of interventions to reduce urinary tract infection in nursing home residents. J Hosp Med. (2017) 12:356–68. doi: 10.12788/jhm.2724, PMID: 28459908 PMC5557395

[85] Wong VWY Huang Y Wei WI Wong SYS Kwok KO. Approaches to multidrug-resistant organism prevention and control in long-term care facilities for older people: a systematic review and meta-analysis. Antimicrob Resist Infect Control. (2022) 11:7. doi: 10.1186/s13756-021-01044-0, PMID: 35033198 PMC8761316

[86] Vlaams Instituut Mondgezondheid: Gezonde Mond (2017–2025). Available online at: https://www.gezondemond.be (Accessed February 2, 2025).

[87] Agentschap Zorg & Gezondheid. Handhygiënecampagne woonzorgcentra (2023). Available online at: https://www.zorg-en-gezondheid.be/sites/default/files/2023-05/Implementatiegids%20Handhygi%C3%ABnecampagne%202023_finaal.pdf (Accessed February 2, 2025).

[88] Departement Zorg. (2023). CAMPAGNE URINEWEGINFECTIES WOONZORGCENTRA. Available online at: https://publicaties.vlaanderen.be/view-file/64476 (Accessed February 2, 2025).

[89] Willemsen I Kluytmans J. The infection risk scan (IRIS): standardization and transparency in infection control and antimicrobial use. Antimicrob Resist Infect Control. (2018) 7:38. doi: 10.1186/s13756-018-0319-z, PMID: 29541449 PMC5845162

[90] Rhee SM Stone ND. Antimicrobial stewardship in long-term care facilities. Infect Dis Clin N Am. (2014) 28:237–46. doi: 10.1016/j.idc.2014.01.001, PMID: 24857390

[91] Centers for Disease Control and Prevention: The core elements of antibiotic stewardship for nursing homes (2015). Available online at: https://www.cdc.gov/antibiotic-use/media/pdfs/core-elements-antibiotic-stewardship-508.pdf (Accessed February 2, 2025).

[92] Westphal GA Lino AS. Systematic screening is essential for early diagnosis of severe sepsis and septic shock. Rev Bras Ter Intensiva. (2015) 27:96–101. doi: 10.5935/0103-507X.20150018, PMID: 26340147 PMC4489775

[93] Chimenti C Sears G McIntyre J. Sepsis in home health care: screening, education, and rapid triage. J Nurs Care Qual. (2021) 36:210–6. doi: 10.1097/NCQ.0000000000000525, PMID: 33079820

[94] Scheer C Giamarellos-Bourboulis E Ferrer R Idelevich E Annane D Artigas A. Sepsis care, diagnostics and quality management: a multidisciplinary cross-sectional survey in 73 countries. The Lancet. (2023). doi: 10.2139/ssrn.4538229

[95] Oanesa RD Su TW-H Weissman A. Evidence for use of validated Sepsis screening tools in the prehospital population: a scoping review. Prehosp Emerg Care. (2024) 28:485–93. doi: 10.1080/10903127.2023.2224862, PMID: 37327065

[96] Scott LJ Redmond NM Tavaré A Little H Srivastava S Pullyblank A. Association between National Early Warning Scores in primary care and clinical outcomes: an observational study in UK primary and secondary care. Br J Gen Pract. (2020) 70:e374–80. doi: 10.3399/bjgp20X709337, PMID: 32253189 PMC7141816

[97] Finnikin S Wilke V. What's behind the NEWS? National Early Warning Scores in primary care. Br J Gen Pract. (2020) 70:272–3. doi: 10.3399/bjgp20X709361, PMID: 32269041 PMC7194012

[98] Mylotte JM. What is the role of nursing homes in the surviving Sepsis campaign? J Am Med Dir Assoc. (2020) 21:41–5. doi: 10.1016/j.jamda.2019.07.022, PMID: 31537482

[99] Sloane PD Ward K Weber DJ Kistler CE Brown B Davis K. Can Sepsis be detected in the nursing home prior to the need for hospital transfer? J Am Med Dir Assoc. (2018) 19:492–6.e1. doi: 10.1016/j.jamda.2018.02.001, PMID: 29599052

[100] Barker RO Stocker R Russell S Roberts A Kingston A Adamson J. Distribution of the National Early Warning Score (NEWS) in care home residents. Age Ageing. (2019) 49:141–5. doi: 10.1093/ageing/afz130, PMID: 31813952 PMC6911654

[101] Dewitte K Scheurwegs E Van Ierssel S Jansens H Dams K Roelant E. Audit of a computerized version of the Manchester triage system and a SIRS-based system for the detection of sepsis at triage in the emergency department. Int J Emerg Med. (2022) 15:67. doi: 10.1186/s12245-022-00472-y, PMID: 36513965 PMC9745734

[102] Keep JW Messmer AS Sladden R Burrell N Pinate R Tunnicliff M. National early warning score at emergency department triage may allow earlier identification of patients with severe sepsis and septic shock: a retrospective observational study. Emerg Med J. (2016) 33:37–41. doi: 10.1136/emermed-2014-204465, PMID: 25971890

[103] Penno EC Crump JA Baird SJ. Performance requirements to achieve cost-effectiveness of point-of-care tests for Sepsis among patients with febrile illness in low-resource settings. Am J Trop Med Hyg. (2015) 93:841–9. doi: 10.4269/ajtmh.15-0082, PMID: 26195467 PMC4596610

[104] Fleuren LM Klausch TLT Zwager CL Schoonmade LJ Guo T Roggeveen LF. Machine learning for the prediction of sepsis: a systematic review and meta-analysis of diagnostic test accuracy. Intensive Care Med. (2020) 46:383–400. doi: 10.1007/s00134-019-05872-y, PMID: 31965266 PMC7067741

[105] Yoon JH Pinsky MR Clermont G. Artificial intelligence in critical care medicine. Crit Care. (2022) 26:75. doi: 10.1186/s13054-022-03915-3, PMID: 35337366 PMC8951650

[106] Burdick H Pino E Gabel-Comeau D Gu C Roberts J Le S. Validation of a machine learning algorithm for early severe sepsis prediction: a retrospective study predicting severe sepsis up to 48 h in advance using a diverse dataset from 461 US hospitals. BMC Med Inform Decis Mak. (2020) 20:276. doi: 10.1186/s12911-020-01284-x, PMID: 33109167 PMC7590695

[107] Haegdorens F Lefebvre J Wils C Franck E Van Bogaert P. Combining the nurse intuition patient deterioration scale with the National Early Warning Score provides more net benefit in predicting serious adverse events: a prospective cohort study in medical, surgical, and geriatric wards. Intensive Crit Care Nurs. (2024) 83:103628. doi: 10.1016/j.iccn.2024.103628, PMID: 38244252

[108] Haegdorens F Wils C Franck E. Predicting patient deterioration by nurse intuition: the development and validation of the nurse intuition patient deterioration scale. Int J Nurs Stud. (2023) 142:104467. doi: 10.1016/j.ijnurstu.2023.104467, PMID: 37068418

[109] Rowland BA Motamedi V Michard F Saha AK Khanna AK. Impact of continuous and wireless monitoring of vital signs on clinical outcomes: a propensity-matched observational study of surgical ward patients. Br J Anaesth. (2024) 132:519–27. doi: 10.1016/j.bja.2023.11.040, PMID: 38135523

[110] Leenen JPL Rasing HJM Kalkman CJ Schoonhoven L Patijn GA. Process evaluation of a wireless wearable continuous vital signs monitoring intervention in 2 general hospital wards: mixed methods study. JMIR Nurs. (2023) 6:e44061. doi: 10.2196/44061, PMID: 37140977 PMC10196902

[111] Nissen SK Candel BGJ Nickel CH de Jonge E Ryg J Bogh SB. The impact of age on predictive performance of National Early Warning Score at arrival to emergency departments: development and external validation. Ann Emerg Med. (2022) 79:354–63. doi: 10.1016/j.annemergmed.2021.09.434, PMID: 34742589

[112] Van de Voorde P Turner NM Djakow J de Lucas N Martinez-Mejias A Biarent D. European resuscitation council guidelines 2021: Paediatric life support. Resuscitation. (2021) 161:327–87. doi: 10.1016/j.resuscitation.2021.02.015, PMID: 33773830

[113] Corfield AR Silcock D Clerihew L Kelly P Stewart E Staines H. Paediatric early warning scores are predictors of adverse outcome in the pre-hospital setting: a national cohort study. Resuscitation. (2018) 133:153–9. doi: 10.1016/j.resuscitation.2018.10.010, PMID: 30336232

[114] Roland D Munro A. Time for paediatrics to screen out sepsis "screening". BMJ. (2023) 381:1327. doi: 10.1136/bmj.p1327, PMID: 37295800

[115] Schlapbach LJ Watson RS Sorce LR Argent AC Menon K Hall MW. International consensus criteria for pediatric Sepsis and septic shock. JAMA. (2024) 331:665–74. doi: 10.1001/jama.2024.0179, PMID: 38245889 PMC10900966

[116] de Groot B Stolwijk F Warmerdam M Lucke JA Singh GK Abbas M. The most commonly used disease severity scores are inappropriate for risk stratification of older emergency department sepsis patients: an observational multi-Centre study. Scand J Trauma Resusc Emerg Med. (2017) 25:91. doi: 10.1186/s13049-017-0436-3, PMID: 28893325 PMC5594503

[117] Mitsunaga T Hasegawa I Uzura M Okuno K Otani K Ohtaki Y. Comparison of the National Early Warning Score (NEWS) and the modified early warning score (MEWS) for predicting admission and in-hospital mortality in elderly patients in the pre-hospital setting and in the emergency department. PeerJ. (2019) 7:e6947. doi: 10.7717/peerj.6947, PMID: 31143553 PMC6526008

[118] Søreide E Morrison L Hillman K Monsieurs K Sunde K Zideman D. The formula for survival in resuscitation. Resuscitation. (2013) 84:1487–93. doi: 10.1016/j.resuscitation.2013.07.020, PMID: 23917078

[119] Semeraro F Greif R Böttiger BW Burkart R Cimpoesu D Georgiou M. European resuscitation council guidelines 2021: systems saving lives. Resuscitation. (2021) 161:80–97. doi: 10.1016/j.resuscitation.2021.02.008, PMID: 33773834

[120] Pepper DJ Sun J Cui X Welsh J Natanson C Eichacker PQ. Antibiotic- and fluid-focused bundles potentially improve Sepsis management, but high-quality evidence is lacking for the specificity required in the centers for Medicare and Medicaid service's Sepsis bundle (SEP-1). Crit Care Med. (2019) 47:1290–300. doi: 10.1097/CCM.0000000000003892, PMID: 31369426 PMC10802116

[121] Daniels R Foot E Pittaway S Urzi S Favry A Miller M. Survey of adherence to sepsis care bundles in six European countries shows low adherence and possible patient risk. BMJ Open Qual. (2023) 12:e002304. doi: 10.1136/bmjoq-2023-002304, PMID: 37286298 PMC10254959

[122] Ye E Ye H Wang S Fang X. Initiation timing of vasopressor in patients with septic shock: a systematic review and META-analysis. Shock. (2023) 60:627–36. doi: 10.1097/SHK.0000000000002214, PMID: 37695641

[123] Stichting Werkgroep Antibioticabeleid (SWAB): The Dutch working party on antibiotic policy (SWAB) guideline for empirical antibacterial therapy of sepsis in adults (2020). Available online at: https://swab.nl/exec/file/download/144 (Accessed February 2, 2025).

[124] Fidalgo P Nora D Coelho L Povoa P. Pancreatic Stone protein: review of a new biomarker in Sepsis. J Clin Med. (2022) 11:1085. doi: 10.3390/jcm11041085, PMID: 35207355 PMC8880320

[125] Van Hecke O Raymond M Lee JJ Turner P Goyder CR Verbakel JY. *In-vitro* diagnostic point-of-care tests in paediatric ambulatory care: a systematic review and meta-analysis. PLoS One. (2020) 15:e0235605. doi: 10.1371/journal.pone.0235605, PMID: 32628707 PMC7337322

[126] Horriar L Rott N Böttiger BW. Improving survival after cardiac arrest in Europe: the synergetic effect of rescue chain strategies. Resusc Plus. (2024) 17:100533. doi: 10.1016/j.resplu.2023.100533, PMID: 38205146 PMC10776426

[127] De Pascale G Antonelli M Deschepper M Arvaniti K Blot K Brown BC. Poor timing and failure of source control are risk factors for mortality in critically ill patients with secondary peritonitis. Intensive Care Med. (2022) 48:1593–606. doi: 10.1007/s00134-022-06883-y, PMID: 36151335

[128] Nguyen YL Wallace DJ Yordanov Y Trinquart L Blomkvist J Angus DC. The volume-outcome relationship in critical care: a systematic review and Meta-analysis. Chest. (2015) 148:79–92. doi: 10.1378/chest.14-2195, PMID: 25927593 PMC4493880

[129] Wilks K Mason D Rice M Seaton R Redpath L Gibbons K. Impact of 1-hour and 3-hour sepsis time bundles on antibiotic use in emergency departments in Queensland, Australia: a before-and-after cohort study. BMJ Open. (2023) 13:e072167. doi: 10.1136/bmjopen-2023-072167, PMID: 37669847 PMC10481845

[130] McKenzie KE Mayorga ME Miller KE Singh N Arnold RC Romero-Brufau S. Notice to comply: a systematic review of clinician compliance with guidelines surrounding acute hospital-based infection management. Am J Infect Control. (2020) 48:940–7. doi: 10.1016/j.ajic.2020.02.006, PMID: 32192754

[131] Gugelmin-Almeida D Tobase L Maconochie I Polastri T Rodrigues Gesteira EC Williams J. What can be learned from the literature about intervals and strategies for paediatric CPR retraining of healthcare professionals? A scoping review of literature. Resusc Plus. (2022) 12:100319. doi: 10.1016/j.resplu.2022.100319 36337082 PMC9630773

[132] Gawad N Allen M Fowler A. Decay of competence with extended research absences during residency training: a scoping review. Cureus. (2019) 11:e5971. doi: 10.7759/cureus.5971, PMID: 31803553 PMC6874279

[133] Damiani E Donati A Serafini G Rinaldi L Adrario E Pelaia P. Effect of performance improvement programs on compliance with sepsis bundles and mortality: a systematic review and meta-analysis of observational studies. PLoS One. (2015) 10:e0125827. doi: 10.1371/journal.pone.0125827, PMID: 25946168 PMC4422717

[134] Burrell AR McLaws ML Fullick M Sullivan RB Sindhusake D. Sepsis kills: early intervention saves lives. Med J Aust. (2016) 204:73. doi: 10.5694/mja15.00657, PMID: 26821106

[135] Kabil G Frost SA Hatcher D Shetty A Foster J McNally S. Early fluid bolus in adults with sepsis in the emergency department: a systematic review, meta-analysis and narrative synthesis. BMC Emerg Med. (2022) 22:3. doi: 10.1186/s12873-021-00558-5, PMID: 35016638 PMC8753824

[136] Shin TG Jo IJ Choi DJ Kang MJ Jeon K Suh GY. The adverse effect of emergency department crowding on compliance with the resuscitation bundle in the management of severe sepsis and septic shock. Crit Care. (2013) 17:R224. doi: 10.1186/cc13047, PMID: 24093643 PMC4055965

[137] Nelson JL Smith BL Jared JD Younger JG. Prospective trial of real-time electronic surveillance to expedite early care of severe sepsis. Ann Emerg Med. (2011) 57:500–4. doi: 10.1016/j.annemergmed.2010.12.008, PMID: 21227543

[138] Doerfler ME D'Angelo J Jacobsen D Jarrett MP Kabcenell AI Masick KD. Methods for reducing sepsis mortality in emergency departments and inpatient units. Jt Comm J Qual Patient Saf. (2015) 41:205–11. doi: 10.1016/S1553-7250(15)41027-X, PMID: 25977247

[139] Mostel Z Perl A Marck M Mehdi SF Lowell B Bathija S. Post-sepsis syndrome - an evolving entity that afflicts survivors of sepsis. Mol Med. (2019) 26:6. doi: 10.1186/s10020-019-0132-z, PMID: 31892321 PMC6938630

[140] Vanhorebeek I Gunst J Casaer MP Derese I Derde S Pauwels L. Skeletal muscle Myokine expression in critical illness, association with outcome and impact of therapeutic interventions. J Endocr Soc. (2023) 7:bvad001. doi: 10.1210/jendso/bvad001, PMID: 36726836 PMC9879715

[141] van der Slikke EC An AY Hancock REW Bouma HR. Exploring the pathophysiology of post-sepsis syndrome to identify therapeutic opportunities. EBioMedicine. (2020) 61:103044. doi: 10.1016/j.ebiom.2020.103044, PMID: 33039713 PMC7544455

[142] Needham DM Davidson J Cohen H Hopkins RO Weinert C Wunsch H. Improving long-term outcomes after discharge from intensive care unit: report from a stakeholders' conference. Crit Care Med. (2012) 40:502–9. doi: 10.1097/CCM.0b013e318232da75, PMID: 21946660

[143] Arero AG Vasheghani-Farahani A Tigabu BM Arero G Ayene BY Soltani D. Long-term risk and predictors of cerebrovascular events following sepsis hospitalization: a systematic review and meta-analysis. Front Med (Lausanne). (2022) 9:1065476. doi: 10.3389/fmed.2022.1065476, PMID: 36507522 PMC9732021

[144] Iwashyna TJ Ely EW Smith DM Langa KM. Long-term cognitive impairment and functional disability among survivors of severe sepsis. JAMA. (2010) 304:1787–94. doi: 10.1001/jama.2010.1553, PMID: 20978258 PMC3345288

[145] Parry SM Granger CL Berney S Jones J Beach L El-Ansary D. Assessment of impairment and activity limitations in the critically ill: a systematic review of measurement instruments and their clinimetric properties. Intensive Care Med. (2015) 41:744–62. doi: 10.1007/s00134-015-3672-x, PMID: 25652888

[146] Bureau C Van Hollebeke M Dres M. Managing respiratory muscle weakness during weaning from invasive ventilation. Eur Respir Rev. (2023) 32:220205. doi: 10.1183/16000617.0205-2022, PMID: 37019456 PMC10074167

[147] Stallmach A Kesselmeier M Bauer M Gramlich J Finke K Fischer A. Comparison of fatigue, cognitive dysfunction and psychological disorders in post-COVID patients and patients after sepsis: is there a specific constellation? Infection. (2022) 50:661–9. doi: 10.1007/s15010-021-01733-3, PMID: 34997542 PMC8741139

[148] Hongo T Yumoto T Naito H Fujiwara T Kondo J Nozaki S. Frequency, associated factors, and associated outcomes of dysphagia following sepsis. Aust Crit Care. (2023) 36:521–7. doi: 10.1016/j.aucc.2022.06.003, PMID: 35851194

[149] Zielske J Bohne S Brunkhorst FM Axer H Guntinas-Lichius O. Acute and long-term dysphagia in critically ill patients with severe sepsis: results of a prospective controlled observational study. Eur Arch Otorrinolaringol. (2014) 271:3085–93. doi: 10.1007/s00405-014-3148-6, PMID: 24970291

[150] Calsavara AJC Nobre V Barichello T Teixeira AL. Post-sepsis cognitive impairment and associated risk factors: a systematic review. Aust Crit Care. (2018) 31:242–53. doi: 10.1016/j.aucc.2017.06.001, PMID: 28645546

[151] Barichello T Sayana P Giridharan VV Arumanayagam AS Narendran B Della Giustina A. Long-term cognitive outcomes after Sepsis: a translational systematic review. Mol Neurobiol. (2019) 56:186–251. doi: 10.1007/s12035-018-1048-2, PMID: 29687346

[152] Luijks ECN van der Slikke EC van Zanten ARH Ter Maaten JC Postma MJ Hilderink HBM. Societal costs of sepsis in the Netherlands. Crit Care. (2024) 28:29. doi: 10.1186/s13054-024-04816-3, PMID: 38254226 PMC10802003

[153] Prescott HC Angus DC. Enhancing recovery from Sepsis: a review. JAMA. (2018) 319:62–75. doi: 10.1001/jama.2017.17687, PMID: 29297082 PMC5839473

[154] Yende S Iwashyna TJ Angus DC. Interplay between sepsis and chronic health. Trends Mol Med. (2014) 20:234–8. doi: 10.1016/j.molmed.2014.02.005, PMID: 24636941 PMC4016696

[155] LUMC (Nederland): TNO Preventie en Gezondheid. Meetinstrumenten in de zorg: TNO-AZL adult quality of life (1999). https://meetinstrumentenzorg.nl/wp-content/uploads/instrumenten/TAAQOL-meetinstr.pdf (Accessed February 2, 2025).

[156] Belgian Health Care Knowledge Centre (KCE). Report on post-intensive care syndrome (PICS) for general practitioners (2020). https://kce.fgov.be/en/publications/all-reports/report-on-post-intensive-care-syndrome-pics-for-general-practitioners (Accessed February 2, 2025).

[157] National Institute for Health and Care Excellence (NICE). Quality standard [QS158]: rehabilitation after critical illness in adults (2017). Available online at: https://www.nice.org.uk/guidance/qs158 (Accessed February 2, 2025).

[158] Guerra WF Mayfield TR Meyers MS Clouatre AE Riccio JC. Early detection and treatment of patients with severe sepsis by prehospital personnel. J Emerg Med. (2013) 44:1116–25. doi: 10.1016/j.jemermed.2012.11.003, PMID: 23321295

[159] Álvarez EA Garrido MA Tobar EA Prieto SA Vergara SO Briceño CD. Occupational therapy for delirium management in elderly patients without mechanical ventilation in an intensive care unit: a pilot randomized clinical trial. J Crit Care. (2017) 37:85–90. doi: 10.1016/j.jcrc.2016.09.002, PMID: 27660922

[160] Weinreich M Herman J Dickason S Mayo H. Occupational therapy in the intensive care unit: a systematic review. Occup Ther Health Care. (2017) 31:205–13. doi: 10.1080/07380577.2017.1340690, PMID: 28692383

[161] Morris PE Goad A Thompson C Taylor K Harry B Passmore L. Early intensive care unit mobility therapy in the treatment of acute respiratory failure. Crit Care Med. (2008) 36:2238–43. doi: 10.1097/CCM.0b013e318180b90e, PMID: 18596631

[162] Zuercher P Moret CS Dziewas R Schefold JC. Dysphagia in the intensive care unit: epidemiology, mechanisms, and clinical management. Crit Care. (2019) 23:103. doi: 10.1186/s13054-019-2400-2, PMID: 30922363 PMC6438038

[163] Hongo T Yamamoto R Liu K Yaguchi T Dote H Saito R. Association between timing of speech and language therapy initiation and outcomes among post-extubation dysphagia patients: a multicenter retrospective cohort study. Crit Care. (2022) 26:98. doi: 10.1186/s13054-022-03974-6, PMID: 35395802 PMC8991938

[164] Guidet B Vallet H Flaatten H Joynt G Bagshaw SM Leaver SK. The trajectory of very old critically ill patients. Intensive Care Med. (2024) 50:181–94. doi: 10.1007/s00134-023-07298-z, PMID: 38236292

[165] Ibarz M Haas LEM Ceccato A Artigas A. The critically ill older patient with sepsis: a narrative review. Ann Intensive Care. (2024) 14:6. doi: 10.1186/s13613-023-01233-7, PMID: 38200360 PMC10781658

[166] Goodacre S Fuller G Conroy S Hendrikse C. Diagnosis and management of sepsis in the older adult. BMJ. (2023) 382:e075585. doi: 10.1136/bmj-2023-075585, PMID: 37451817

[167] Fitzgerald JC Kelly NA Hickey C Balamuth F Thomas NH Hogan A. Implementation of a follow-up system for pediatric Sepsis survivors in a large academic pediatric intensive care unit. Front Pediatr. (2021) 9:691692. doi: 10.3389/fped.2021.691692, PMID: 34150690 PMC8212949

[168] Raman S English A O'Keefe M Harley A Steele M Minogue J. Designing support structures post Sepsis in children: perspectives of the Queensland Paediatric Sepsis program. Front Pediatr. (2021) 9:759234. doi: 10.3389/fped.2021.759234, PMID: 34869116 PMC8636900

[169] Weiss SL Fitzgerald JC Pappachan J Wheeler D Jaramillo-Bustamante JC Salloo A. Global epidemiology of pediatric severe sepsis: the sepsis prevalence, outcomes, and therapies study. Am J Respir Crit Care Med. (2015) 191:1147–57. doi: 10.1164/rccm.201412-2323OC, PMID: 25734408 PMC4451622

[170] Eijkholt M. Medicine's collision with false Hope: the false Hope harms (FHH) argument. Bioethics. (2020) 34:703–11. doi: 10.1111/bioe.12731, PMID: 32134519 PMC7664828

[171] Philippart F Vesin A Bruel C Kpodji A Durand-Gasselin B Garçon P. The ETHICA study (part I): elderly's thoughts about intensive care unit admission for life-sustaining treatments. Intensive Care Med. (2013) 39:1565–73. doi: 10.1007/s00134-013-2976-y, PMID: 23765236

[172] Le Guen J Boumendil A Guidet B Corvol A Saint-Jean O Somme D. Are elderly patients' opinions sought before admission to an intensive care unit? Results of the ICE-CUB study. Age Ageing. (2016) 45:303–9. doi: 10.1093/ageing/afv191, PMID: 26758531

[173] Lee HY Lee J Jung YS Kwon WY Oh DK Park MH. Preexisting clinical frailty is associated with worse clinical outcomes in patients with Sepsis. Crit Care Med. (2022) 50:780–90. doi: 10.1097/CCM.0000000000005360, PMID: 34612849

[174] Guidet B de Lange DW Boumendil A Leaver S Watson X Boulanger C. The contribution of frailty, cognition, activity of daily life and comorbidities on outcome in acutely admitted patients over 80 years in European ICUs: the VIP2 study. Intensive Care Med. (2020) 46:57–69. doi: 10.1007/s00134-019-05853-1, PMID: 31784798 PMC7223711

[175] Ibarz M Boumendil A Haas LEM Irazabal M Flaatten H de Lange DW. Sepsis at ICU admission does not decrease 30-day survival in very old patients: a post-hoc analysis of the VIP1 multinational cohort study. Ann Intensive Care. (2020) 10:56. doi: 10.1186/s13613-020-00672-w, PMID: 32406016 PMC7221097

[176] Matsuda W Yamamoto M. How should we use frailty evaluation for patients with Sepsis in the clinical practice? Crit Care Med. (2022) 50:e399–400. doi: 10.1097/CCM.0000000000005417, PMID: 35311786

[177] Darvall JN Bellomo R Paul E Subramaniam A Santamaria JD Bagshaw SM. Frailty in very old critically ill patients in Australia and New Zealand: a population-based cohort study. Med J Aust. (2019) 211:318–23. doi: 10.5694/mja2.50329, PMID: 31489652

[178] Wang AY Chang CK. Do-not-attempt resuscitation independently predict in-hospital mortality in septic patients. Am J Emerg Med. (2020) 38:953–7. doi: 10.1016/j.ajem.2019.158362, PMID: 31358382

[179] Chang YC Fang YT Chen HC Lin CY Chang YP Chen YM. Effect of do-not-resuscitate orders on patients with sepsis in the medical intensive care unit: a retrospective, observational and propensity score-matched study in a tertiary referral hospital in Taiwan. BMJ Open. (2019) 9:e029041. doi: 10.1136/bmjopen-2019-029041, PMID: 31209094 PMC6589004

[180] Zhang Z Hong Y Liu N Chen Y. Association of do-not-resuscitate order and survival in patients with severe sepsis and/or septic shock. Intensive Care Med. (2017) 43:715–7. doi: 10.1007/s00134-017-4690-7, PMID: 28154886

[181] Sarkari NN Perman SM Ginde AA. Impact of early do-not-attempt-resuscitation orders on procedures and outcomes of severe sepsis. J Crit Care. (2016) 36:134–9. doi: 10.1016/j.jcrc.2016.06.030, PMID: 27546762 PMC5967875

[182] Baldwin MR Narain WR Wunsch H Schluger NW Cooke JT Maurer MS. A prognostic model for 6-month mortality in elderly survivors of critical illness. Chest. (2013) 143:910–9. doi: 10.1378/chest.12-1668, PMID: 23632902 PMC3616685

[183] Haas LEM Boumendil A Flaatten H Guidet B Ibarz M Jung C. Frailty is associated with long-term outcome in patients with sepsis who are over 80 years old: results from an observational study in 241 European ICUs. Age Ageing. (2021) 50:1719–27. doi: 10.1093/ageing/afab036, PMID: 33744918

[184] Hanson S Brabrand M Lassen AT Ryg J Nielsen DS. What matters at the end of life: a qualitative study of older peoples perspectives in southern Denmark. Gerontol Geriatr Med. (2019) 5:2333721419830198. doi: 10.1177/2333721419830198, PMID: 30815513 PMC6381425

[185] Cuijpers ACM Coolsen MME Schnabel RM Lubbers T van der Horst ICC van Santen S. Self-perceived recovery and quality of life in elderly patients surviving ICU-admission for abdominal sepsis. J Intensive Care Med. (2022) 37:970–8. doi: 10.1177/08850666211052460, PMID: 34756128 PMC9136475

[186] Khoujah D Martinelli AN Winters ME. Resuscitating the critically ill geriatric emergency department patient. Emerg Med Clin North Am. (2019) 37:569–81. doi: 10.1016/j.emc.2019.04.002, PMID: 31262422

[187] Barbash IJ. Moving forward: frailty and adverse Sepsis outcomes. Crit Care Med. (2022) 50:880–2. doi: 10.1097/CCM.0000000000005377, PMID: 35485588

[188] Komori A Abe T Yamakawa K Ogura H Kushimoto S Saitoh D. Characteristics and outcomes of frail patients with suspected infection in intensive care units: a descriptive analysis from a multicenter cohort study. BMC Geriatr. (2020) 20:485. doi: 10.1186/s12877-020-01893-1, PMID: 33218303 PMC7677099

[189] Mentzelopoulos SD Couper K Voorde PV Druwé P Blom M Perkins GD. European resuscitation council guidelines 2021: ethics of resuscitation and end of life decisions. Resuscitation. (2021) 161:408–32. doi: 10.1016/j.resuscitation.2021.02.017, PMID: 33773832

[190] Brummel NE Bell SP Girard TD Pandharipande PP Jackson JC Morandi A. Frailty and subsequent disability and mortality among patients with critical illness. Am J Respir Crit Care Med. (2017) 196:64–72. doi: 10.1164/rccm.201605-0939OC, PMID: 27922747 PMC5519959

[191] Somogyi-Zalud E Zhong Z Lynn J Dawson NV Hamel MB Desbiens NA. Dying with acute respiratory failure or multiple organ system failure with sepsis. J Am Geriatr Soc. (2000) 48:S140–5. doi: 10.1111/j.1532-5415.2000.tb03123.x, PMID: 10809467

[192] Chang DW Brass EP. Patient and hospital-level characteristics associated with the use of do-not-resuscitate orders in patients hospitalized for sepsis. J Gen Intern Med. (2014) 29:1256–62. doi: 10.1007/s11606-014-2906-x, PMID: 24928264 PMC4139525

[193] Knight T Malyon A Fritz Z Subbe C Cooksley T Holland M. Advance care planning in patients referred to hospital for acute medical care: results of a national day of care survey. EClinicalMedicine. (2020) 19:100235. doi: 10.1016/j.eclinm.2019.12.005, PMID: 32055788 PMC7005412

[194] Mason B Boyd K Steyn J Kendall M Macpherson S Murray SA. Computer screening for palliative care needs in primary care: a mixed-methods study. Br J Gen Pract. (2018) 68:e360–9. doi: 10.3399/bjgp18X695729, PMID: 29581129 PMC5916083

[195] Street M Ottmann G Johnstone MJ Considine J Livingston PM. Advance care planning for older people in Australia presenting to the emergency department from the community or residential aged care facilities. Health Soc Care Community. (2015) 23:513–22. doi: 10.1111/hsc.12162, PMID: 25443161

[196] Sampson EL Jones L Thuné-Boyle IC Kukkastenvehmas R King M Leurent B. Palliative assessment and advance care planning in severe dementia: an exploratory randomized controlled trial of a complex intervention. Palliat Med. (2011) 25:197–209. doi: 10.1177/0269216310391691, PMID: 21228087

[197] Dwyer R Stoelwinder J Gabbe B Lowthian J. Unplanned transfer to emergency departments for frail elderly residents of aged care facilities: a review of patient and organizational factors. J Am Med Dir Assoc. (2015) 16:551–62. doi: 10.1016/j.jamda.2015.03.007, PMID: 25933726

[198] Oulton J Rhodes SM Howe C Fain MJ Mohler MJ. Advance directives for older adults in the emergency department: a systematic review. J Palliat Med. (2015) 18:500–5. doi: 10.1089/jpm.2014.0368, PMID: 25763860

[199] Vlaams Instituut voor Kwaliteit van Zorg: VLAAMS INDICATORENPROJECT WOONZORGCENTRA: KWALITEITSINDICATOREN (2022). Available online at: https://www.zorgkwaliteit.be/sites/default/files/2024-01/Publieksrapport2022_def.pdf (Accessed February 2, 2025).

[200] Zisberg A Shadmi E Gur-Yaish N Tonkikh O Sinoff G. Hospital-associated functional decline: the role of hospitalization processes beyond individual risk factors. J Am Geriatr Soc. (2015) 63:55–62. doi: 10.1111/jgs.13193, PMID: 25597557

[201] Lemoyne SE Herbots HH De Blick D Remmen R Monsieurs KG Van Bogaert P. Appropriateness of transferring nursing home residents to emergency departments: a systematic review. BMC Geriatr. (2019) 19:17. doi: 10.1186/s12877-019-1028-z, PMID: 30665362 PMC6341611

[202] Caplan GA Coconis J Woods J. Effect of hospital in the home treatment on physical and cognitive function: a randomized controlled trial. J Gerontol A Biol Sci Med Sci. (2005) 60:1035–8. doi: 10.1093/gerona/60.8.1035, PMID: 16127109

[203] Edgar K Iliffe S Doll HA Clarke MJ Gonçalves-Bradley DC Wong E. Admission avoidance hospital at home. Cochrane Database Syst Rev. (2024) 3:Cd007491. doi: 10.1002/14651858.CD007491.pub2 38438116 PMC10911897

[204] European Centre for Disease Prevention and Control: Point prevalence survey of healthcare-associated infections and antimicrobial use in European acute care hospitals −2022-2023 (2024). Available online at: https://www.ecdc.europa.eu/en/publications-data/PPS-HAI-AMR-acute-care-europe-2022-2023 (Accessed February 2, 2025).

[205] Monitoring Intensive Care Activities (MICA): Belgisch Nationaal ICU register (2020). Available online at: https://www.micaprogram.be/nl/ (Accessed February 2, 2025).

[206] Belgian Federal Public Service Health, Food chain Safety and Environment. Belgian sepsis national action plan (2024). Available online at: https://overlegorganen.gezondheid.belgie.be/sites/default/files/documents/belgian_sepsis_national_action_plan_be-snap_v1_30052024_f.pdf (Accessed February 2, 2025).

