# Supplementary material for: Be-SNAP: the Belgian Sepsis National Action Plan
Source: Front Public Health. 2025 Jul 1;13:1575502. doi: 10.3389/fpubh.2025.1575502 (PMC12259572; doi:10.3389/fpubh.2025.1575502)
Supplement: Supplementary file 1 [file Table_1.docx]

Supplementary Material

# Table Overview of actionable recommendations.

| Aim | Main Actors | Additional considerations (acceptable, affordable, effective…?) |
| --- | --- | --- |
| 1. Awareness and knowledge on sepsis | | |
| 1.1 Create a National Sepsis Forum (a) and a National Sepsis Foundation (b) | | |
| (a) to coordinate all initiatives  (b) to advocate for ‘sepsis’ policy making & provide peer support, education. | (a) should be an interfederal, funded structure, that includes public health officers, scientific experts, (para)medical professionals & representatives of patient advocacy groups.  (b) will be a private initiative with mixed public and private funding, like for instance the Diabetes Liga / Forum. Building up from already existing patient advocacy groups (e.g. Sepsibel). | Such structures already exist in other countries or for other medical problems in Belgium. There could be synergies with existing structures that would facilitate the process. Central coordination is anyhow vital for overseeing activities across various competencies. Importantly, these structures guarantee long-term sustainability. Overall costs are limited and mostly concern staffing. Funding is primarily governmental, but can also come in part from private sources. |
| 1.2 Design (a) a general awareness campaign & (b) a focused campaign for high-risk groups | | |
| (a) for the public, ‘branding’ sepsis.  (b) for *high-risk groups* & for those with limited health information seeking | Central role for the National Sepsis Forum & the Foundation, in collaboration with several existing actors such as communication services of regional and federal public health administrations, scientific societies, healthcare professionals, partners involved in infection prevention….  Partnerships with private companies e.g. industry, insurance companies... | A lot of material / examples exist from other countries – international campaigns (e.g. World sepsis Day 13/09). Existing platforms, websites, apps can be used. Survivor testimonials can be of value. Overall, the cost for this intervention compared to the potential gain is low. There is a need for an overall strategy and coordination with existing campaigns about appropriate antimicrobial use and medical resource use (e.g. emergency department consultation). |
| 1.3 Provide tailored educational packages for all relevant healthcare professionals | | |
| Tailored to their specific needs, and in view of the patients they take care of (including e.g. children, elderly, immune depressed).  Promote awareness - focus on recognition and early adequate management, prudent use of antibiotics, infection prevention, but also long-term impact, advanced care planning. | Professional societies, universities and other higher education centres, in collaboration with the National Sepsis Forum. | A lot of material exists from other countries but needs to be adapted to the local healthcare system. Although well-studied, there might be a lack of consensus about certain advice and actions. International guidelines need to be evaluated for Belgian consensus and subsequent use.  Incorporation into existing teaching curricula demands close collaboration with educational actors and policy makers. Integration in life-long learning initiatives (accreditation, congresses etc) is the combined responsibility of scientific organisations, RIZIV/INAMI and other actors involved in this. |
| 2. Prevention of sepsis and safeguarding treatment options | | |
| 2.1 Prevent sepsis & safeguard antibiotics' effectiveness in the community | | |
| Improve health literacy & access to (chronic) healthcare -including for instance dental care, wound care…- for all, with specific attention to most vulnerable (medically & socio-economically).  Invest in vaccination literacy and vaccination programs, especially for the 65+, medically vulnerable, or pregnant.  Develop specific guidelines for infection prevention at home (incl. food safety) & for specific home care procedures such as for indwelling catheters... | National sepsis foundation in collaboration with communication services of regional public health actors and health institutes.  Strong involvement of among others first line health zones, regional public health administrations, patient advocacy groups, social security…. | Need for tailored information campaigns (see also 1.2) & educational initiatives (see 1.3). At least in part via financial initiatives (government) to lower (or not increase) barriers or facilitate access to care, to vaccination, to information…  Specific need to explore and implement hospital at home practices and develop guidelines for this.  Potential program development via pharmacists or more general 'health houses' can be explored. |
| 2.2 Set up a national multimodal strategy to prevent healthcare-associated Sepsis (in hospitals). Further strengthen already existing AMS activities. | | |
| Further strengthen existing hospital Antibiotic Policy Groups.  Need for freely available, regularly updated, antimicrobial treatment guidelines for hospitals.  Integrate IPC activities in the hospital strategic plan.  Aim for a culture change within the establishment of a ‘safety climate’. | Engagement of all different actors within each hospital, from micro to macro, including specifically hospital management, existing IPC teams….  Also larger, government organisations & policy makers, scientific societies and institutions (e.g. Sciensano <http://www.sciensano.be>). | Aligning strategies with existing recommendations (e.g. KCE, Sciensano) demands consensus. Need to further streamline different existing initiatives about infection prevention, AMS and sepsis prevention. Need for formal staffing standards and sufficient financial support for clinical infectiologists, beyond the current IPC initiatives.  Provision of up-to-date local *guidelines for antimicrobial treatment* is considered of critical importance and very cost-effective. Developing such guidelines should be facilitated by the government, whether or not under the umbrella of the National Focal Point (see 1.1). |
| 2.3 Specifically strengthen IP and AMS in LCTF | | |
| Define specific objectives & quality indicators, as well as guidelines & multimodal strategies to implement them. Define priorities, both for what concerns concrete interventions and for surveillance. Examples include hand hygiene, UTI guidelines…but also improved vaccination coverage for residents and staff, advance care planning, transfer guidelines…  Specifically support the coordinating and advising clinicians ('CRA') and the nurses working in LTCF.  Adapt existing materials to the level and practice of those working in LTCF, including nursing aids, paramedical professionals…. | Engagement of all different actors within each LCTF, from micro to macro, including specifically management, CRA physicians, nurses….  Also larger, government organisations and policy makers, scientific societies and institutions (e.g. Sciensano). | There have already been pilot projects in Belgium that demonstrated feasibility and identified pathways to reach the identified goals. (https://www.health.belgium.be/sites/default/files/uploads/fields/fpshealth_theme_file/verslag_nl.pdf ; in Dutch)  The Flemish government put forward several initiatives to improve IP and AMS, and specifically vaccination coverage and advance care planning in LCTF. However, with limited success till currently. To improve this, there is need for education and data registration/ surveillance, but even more for facilitators. LTCF staff should be empowered (incl. mandate) and supported (incl. financially) to coordinate and implement IPC and AMS practices in their environment. Guidelines can help in this when they are adapted to the concrete reality of each LTCF. A crucial factor in all this *is sufficient staffing*. |
| 3. Early recognition and rapid response systems | | |
| 3.1 Use context-specific, effective EWS for screening, integrated in a broader Rapid Response Strategy. Implement Rapid Response Teams in hospitals. | | |
| Context-specific equally include: Primary care settings, LTCF, pre-hospital teams, emergency departments, hospital wards, intensive care settings…  The National Early Warning Score (NEWS) 1 or 2 currently is considered as the most appropriate early warning score for Belgium. Specific scores for specific subpopulations such as paediatric or geriatric patients.  Evaluate the added use and effectiveness of bedside point of care tests to further improve test performance of scores.  Integrate in a broader strategy of rapid response, via rapid response / medical emergency teams within the hospital and/or standard procedures for alarming and first response both prehospital, in LTCF, as well as in hospital. On a 24/7 365 basis but adapted to local needs. | Engagement of all different actors within each hospital, from micro to macro, including specifically hospital management.  Also larger, government organisations and policy makers, scientific societies and institutions to advice on procedures and provide proper reimbursement. | Even if there will be an added cost to implement such rapid response strategies, the impact of any missed or delayed recognition is such that these measures are currently considered cost-effective.  Conditional for implementation is on the one hand adequate staffing (which is definitely an issue) and on the other hand supportive tools (guidelines, procedures, electronic patient records, artificial intelligence solutions…).  Rapid response teams can provide -24/7 365- proactive, reactive, and supportive care within the hospital, in addition to offering educational support.  Importantly, this support goes beyond the problem of sepsis and is of impact for all pathologies that are acute and timely. |
| 4. Early adequate treatment | | |
| 4.1 Hospitals should guarantee the 24/7 availability of specifically trained ‘advanced’ care teams | | |
| The care provided for each patient when recognised with sepsis should be tailored to the level of knowledge and skills expected from the attending provider. While these are basic for many providers, from certain providers we expect an ability to provide early ‘advanced’ interventions at the bedside outside of the ICU environment.  Specific attention should be given to subpopulations such as children, elderly or those immune-depressed. | Engagement of all different actors within each hospital, from micro to macro, including specifically hospital management. Also larger, government organisations and policy makers, scientific societies and institutions to advice on procedures and provide proper reimbursement. | Clear guidelines about what constitutes proper emergency treatment for sepsis, in view of the capacity of each provider, are needed. Define level of knowledge and skills expected from the attending provider. More specifically, the minimum training and performance requirements for such ‘advanced’ teams should be defined. Governments should provide proper financial support for its implementation (staffing, resource use...) -knowing that again this will be cost-effective in the long term- and thus also regulate it.  On the local level, healthcare systems and hospitals should look at their existing standard operating procedures and if necessary, develop additional ones to clarify indications and communication pathways for basis providers to contact such advanced teams. The teams themselves -within the regulations of the government- can be of different compositions and professional background. |
| 4.2 ICU should have specific, where possible evidence-based, procedures in place to care for all critical sepsis patients – including specific subpopulations. | | |
| Specific patient populations (e.g. children, those with refractory shock or severe organ failure, needing organ support or even extracorporeal membrane oxygenation) might demand early referral to a dedicated highly specialised ICU and each ICU should have plans and collaboration agreements to allow for this. | Healthcare structures with help of scientific and professional societies and National Sepsis Forum. The subsidising government. | The government by means of financial incentives and /or regulatory measures, should acknowledge and facilitate the specific position of tertiary care ICU in referral pathways for specific subpopulations and/or for patients with refractory shock, including the necessary highly- specialised emergency transport.  Consensus between all involved actors about such pathways and what constitute tertiary care ICU and what not, is conditional for this. |
| 5. Post-sepsis care and rehabilitation | | |
| 5.1 Develop a multidisciplinary rehabilitation care pathway for sepsis patients that encompasses the psychological, neurological and physical domains, with clear transition into home setting. | | |
| A pathway means a trajectory of care that should start very early and goes further *beyond* the borders of ICU, hospital or rehabilitation centre. Rehabilitation should be individualised, patient and family-centric, emphasising the patient’s values and preferences but also the interaction between the patient and their close relatives. It should address the psychological, neurological, and physical challenges faced. | Hospitals, professional societies for intensive care, and rehabilitation, first line actors, RIZIV/INAMI. Healthcare professionals at ICUs and rehabilitation services (medical, nursing, physiotherapy/ergotherapy, psychology, social services…).  Survivor networks such as Sepsibel could enhance recovery by reducing isolation and promoting shared experiences.  Integrating post-sepsis rehabilitation pathways into existing chronic care can provide a more streamlined and cost-effective approach. | The concept of multidisciplinary care pathways already exists in Belgium e.g. geriatric, Covid… (and in other countries). There might be overlap with other severe diseases needing prolonged intensive care. More specifically, we also refer to the post-ICU syndrome.  While implementing such programs might be costly, the cost-effectiveness ratio is likely positive given the even higher impact of not implementing them.  Specific attention should be given to the (timely) transition from hospital to a LTCF and/or home and how to facilitate this.  We plea for a holistic approach that also takes into account the impact on those close to the patient, relatives and caretakers. |
| 5.2 Facilitate peer support. | | |
| Encouraging interactions with fellow sepsis survivors.  Acknowledge and support the psychosocial impact of surviving sepsis and providing peer support to diminish ‘sense of loneliness and isolation’. | The Belgian national sepsis Foundation. Healthcare providers in different contexts should be able to refer and connect patients and family with survivors and experts via the foundation. | See 1.1b. Building on the expertise and engagement of the existing patient groups. Provide more structure and coaching to improve on the way these groups work and what they can achieve. |
| 6. Ethical considerations | | |
| 6.1 Provide targeted education of & communication to both HCP & the public about Advance Care Planning. | | |
| Can be specifically focussed on sepsis or rather be part of a larger initiative about ACP and critical illness / resuscitation. The expected outcomes, taking into account patient context and characteristics, should be clarified to all to assist in decision making. ACP typically is ‘in advance’ and is the result of a thorough discussion with the patient and/or his relatives about treatment expectations, values and preferences… | Engagement of all different healthcare providers with direct patient contact, included but not limited to the general practitioner, the hospital specialist, the CRA physician, nurse specialists…. Scientific societies and patient groups to provide guidance. Governments to provide a proper juridical and organisational context. | There is already a Belgian existing legislative and regulatory framework on ACP (e.g. recently included in the new law on patients' rights March 2024), also concordant with Advice N° 84 (27/3/2023) of the National Comity for Bioethics (https://www.health.belgium.be/sites/default/files/uploads/fields/fpshealth_theme_file/230327_advies_84_toegang_tot_zorg_in_wzc.pdf). This goes beyond sepsis, although sepsis is often one of the drivers for worsening condition and thus treatment limitation discussions. There is a direct relationship with initiatives about ‘palliative’ care, which have broadened their target audience and indications to earlier in a patient’s trajectory.  Conditional is knowledge about process and outcomes on the one hand, and on the other hand about the process. This demands education and advocacy. Truly implementing this on a larger scale demands governmental initiative (ACP is a quality indicator for LTCF by the Flemish government, yet this fact did not yet significantly increase its implementation). There are existing materials and even a dedicated governmental website (https://mijnoudedag.be/) that is of good quality but largely unknown. Information campaigns might have a positive impact but only if they are part of a societal debate and larger plan on ‘advance care planning and goals of care’. |
| 6.2 GPs should be supported & stimulated to take up the responsibility to assure proper ACP, at least for all their patients with a high-risk profile for critical illness and/or the frail. | | |
| In the specific setting of residential care facilities and nursing homes, the coordinating physician (CRA), as well as other members of the medical team, can help to identify these patients (which should be part of the measured quality indicators for that facility). | Engagement of all different healthcare providers with direct patient contact. Scientific societies and patient groups to provide guidance. Governments to provide a proper juridical, financial and organisational context. | Only a limited number of frail persons in Belgium have a retrievable ACP, illustrating that there are still important barriers for effective implementation. Reimbursement framework already exists, but not well adapted to reality as there is need for multidisciplinarity, repeated consultation, pre-palliative consulting…. The cost of unintended /undesired/inappropriate critical care in frail patients is far higher so investing in ACP is cost-effective, especially when also taking emotional and psychological outcomes (of patient, relatives and healthcare providers) into equation.  Advanced nurse practitioners could be of great value to help and (re)discuss the topics of ACP in a low threshold manner, linked to specific clinical situations e.g. need for oxygen at home or loss of mobility. Implementing locoregional ethical advisory boards could further support. |
| 6.3 ACP conclusions should be made available 24/7 in existing clear advance directives, for all relevant healthcare providers via electronic way. | | |
| The directive needs to be as explicit as possible on the type of care the person would wish for (standardised content). | Governments to provide a proper juridical, financial and organisational context. IT solutions within public health services. | Not a high cost and crucial for overall implementation of ACP. One could work via the global medical GP files (e.g. Sumehr) or alternative e-health options. GDPR-rules need to be respected at all times. |
| 6.4 Further develop transmural 'hospital at home’ programs at the locoregional level to facilitate proper ACP. | | |
| These programs offer medical assessment, treatment, and follow-up at home for patients who no longer want hospitalisation -or for whom hospitalisation is mostly harmful- yet would still benefit from for instance short-term intravenous antibiotics or oxygen therapy. | Hospitals, first line zones, nursing homes. Governments to provide a proper juridical, financial and organisational context. | Certain transmural initiatives already exist. Experience with these suggest that treatment costs can be up to 10-fold lower at home without increasing risks. Patients’ satisfaction is high for selected populations. A financial framework already exists and that with clear definitions of types of care that can be provided. Broader implementation and structural reimbursement is advisable. International examples such as in the UK may inform this trajectory. |
| 7. Surveillance and research | | |
| 7.1 Establish a Belgian sepsis research coordination centre, whether or not as part of the Sepsis Focal Point | | |
| Such coordination centre can prioritise research, streamline efforts, facilitate collaboration (national and international), and disseminate findings effectively.  A *national sepsis research day* may be organised on a regular basis to increase interest and disseminate results.  Research should include a broad range of relevant topics including socio-epidemiological context, early warning and diagnostics, pathophysiology, therapeutic trials, outcome and public health impact. It should focus on the local public health context. It should specially look at *cost-effectiveness* of certain interventions (e.g. rapid response systems, home monitoring…) for the Belgian healthcare system, as well as at gaps in knowledge and awareness of target groups. | Central role for the National Sepsis Forum & the Foundation, in collaboration with several existing actors such as regional and federal public health administrations and research institutions (KCE, FWO/FNWS, Sciensano), scientific societies, healthcare professionals ….  Partnerships with private companies e.g. industry, insurance companies... .  International collaboration with scientific societies, funding agencies, international research bodies… . | Context & healthcare system specific data are crucial for policy making on both the micro and the macro level. Monitoring progress on the implementation and effectiveness of suggested interventions is an essential part of multimodal strategies. Data about sepsis, when available, can be added to the national health data (https://www.gezondbelgie.be/nl/gezondheidstoestand) and used to support national quality improvement initiatives.  The overall cost for obtaining these sorts of data and implementing such research is relatively limited given their broad potential impact. Cost will depend on the research methodologies chosen (e.g. extension of hospital-based surveillance, use of sentinel hospitals and practices, government-driven mandatory registration…).  Conditional is to coordinate this at a regional/federal level so that research has the optimal scale, focus etc.  To guarantee dedicated resources, sepsis should be identified as a standalone category in funding programs. |
| 7.2 A centralised Belgian sepsis registry should be developed to track all (or at least a representative group) of sepsis cases in Belgium, including those with septic shock, & to collect detailed patient data across all healthcare settings. | | |
| Using a standardised case definition which is the current Sepsis-3 definition for adult patients and the recently published Phoenix criteria for children. Data collection will have to be context specific. It should be kept *as easy as possible* for the healthcare providers involved, using integrating automatic data capture within electronic health records and patient data management systems.  Such data might be integrated in the ongoing point prevalence study (Sciensano) and other monitoring/ surveillance studies in the field of infectious diseases. | Central role for the in 7.1 established Sepsis research coordination centre, in collaboration with the national sepsis Forum and several other existing actors such as regional and federal public health administrations and research institutions (KCE, FWO/FNWS, Sciensano), scientific societies, healthcare professionals.  There is a specific role for Sciensano, as the research institute for the federal government of health, as they already collect registry data on for instance healthcare-associated bloodstream infection. Such registries could be extended and/or linked to other initiatives. | Generating local data is essential for data-informed policy. Care should be given to correct interpretation of such data, as more attention and education may initially lead to an increased number of diagnoses and thus worse outcomes.  As stated above, cost will depend on methodologies used but are considered relatively small compared to their potential use. Data sources could include e.g. extension of hospital-based surveillance of bloodstream infections, use of sentinel hospitals and GP-practices… . |
